# Effects of *APOA5 −1131T>C* (rs662799) on Fasting Plasma Lipids and Risk of Metabolic Syndrome: Evidence from a Case-Control Study in China and a Meta-Analysis

**DOI:** 10.1371/journal.pone.0056216

**Published:** 2013-02-28

**Authors:** Chunxiao Xu, Rongpan Bai, Dandan Zhang, Zhenli Li, Honghong Zhu, Maode Lai, Yimin Zhu

**Affiliations:** 1 Department of Epidemiology & Biostatistics, School of Public Health, Zhejiang University, Hangzhou, Zhejiang, People's Republic of China; 2 Bioelectromagnetics Laboratory, School of Public Health, Zhejiang University, Hangzhou, Zhejiang, People's Republic of China; 3 Department of Pathology and Pathophysiology, School of Medicine, Zhejiang University, Hangzhou, Zhejiang, People's Republic of China; 4 Department of Public Health, College of Health and Human Services, Western Kentucky University, Bowling Green, Kentucky, United States of America; Vanderbilt University, United States of America

## Abstract

The apolipoprotein A5 (*APOA5*) gene *−1131T>C* (rs662799) has been suggested to be involved in the pathway of lipid homeostasis and the development of metabolic syndrome (MetS). However, the findings are not consistent. To systematically evaluate the associations between *−1131T>C* polymorphism and fasting lipid parameters and the risk of MetS, we conducted a case-control study in a Chinese population and a meta-analysis. The findings from 1840 Chinese participants indicated that the C allele carriers had significantly higher fasting total cholesterol (TC), triglycerides (TG) and lower HDL-cholesterol (HDL-C) than the TT homozygotes carriers. The *−1131C* allele was also found to be significantly associated with increased risk of MetS (OR  =  1.40, 95% confidence interval (CI)  =  1.15, 1.69) compared to the TT homozygotes. In the meta-analysis of 51,868 participants from 46 East Asian studies, 26 European studies and 19 studies of other ethnic groups, the *−1131C* allele was associated with higher fasting TC (weighted mean difference (WMD)  =  0.08 mmol/L, 95% CI  =  0.05, 0.10, *P* = 1.74×10^−9^), TG (WMD  =  0.30 mmol/L, 95% CI  =  0.26, 0.33, *P* =  1.87×10^−55^), LDL-cholesterol (LDL-C) (WMD  =  0.04 mmol/L, 95% CI  =  0.02, 0.07, *P* = 0.002), and lower HDL-C (WMD  =  −0.05 mmol/L, 95% CI  =  −0.06,−0.04, *P* = 1.88×10^−21^), respectively. Based on 12 studies with 5,573 MetS cases and 8,290 controls from 5 East Asian studies, 5 European studies and 2 studies of other ethnic groups, the *−1131C* allele was associated with increased risk of MetS with an OR (95% CI)  =  1.33 (1.16, 1.53) in the overall population, 1.43 (1.29, 1.58) in East Asian and 1.30 (0.94, 1.78) in European populations. In conclusion, the *−1131C* allele may be associated with elevated levels of fasting TG, TC, LDL-C and decreased HDL-C, and increased risk of MetS, especially in East Asians.

## Introduction

Metabolic syndrome (MetS), characterized by visceral obesity, dyslipidemia, hypertension and hyperglycemia, has become one of the major public health challenges worldwide [1]. The prevalence of MetS is currently around 30% and is rising worldwide [2]. Besides environmental risk factors such as high energy intake and low physical activity, genetic variation may play a key role in predisposing to MetS. Recent candidate gene and genome-wide association studies (GWAS) have identified a few susceptibility loci for MetS [3]–[4]. The apolipoprotein A5 (*APOA5*) gene has been found to be associated with the increased risk of MetS. The *+1891T>C* (rs2266788) in *APOA5* gene was reported associated with MetS in a European GWAS [3]. Another intergenic locus in *APOA1/C3/A4/A5* gene cluster region, rs964184, was identified associated with MetS in Finnish populations by a GWAS [4]. Therefore, *APOA5* is considered a potential biomarker for MetS.

*APOA5* gene is part of the *APOA1/A4/C3/A5* gene cluster on 11q23, identified by comparative sequencing analysis [5]. The human *APOA5* gene consists of four exons and three introns. It codes a protein with 369 amino acids. The human *APOA5* gene is exclusively expressed in liver and its product apoA-V can be detected in very low-density lipoprotein (VLDL), high-density lipoprotein (HDL) and chylomicrons. It plays an important role in regulating plasma triglyceride levels in both human beings and mice [6]. The *−1131T>C*, in the promoter region of *APOA5* gene, is the most extensively studied variant. The *−1131T>C* has been reported to be associated with hypertriglyceridemia [7]–[10]. The findings, however, are not consistent. No associations of the *−1131T>C* polymorphism with triglycerides (TG) have been reported in African-American or European women by Pennachio and Rubin [11]. The *−1131C* allele has also been reported to be associated with higher low-density lipoprotein-cholesterol (LDL-C) [12] and lower HDL-C levels [9]. Also, the association of this polymorphism with MetS has not been consistently found among different studies and/or different populations. Significant associations have been reported by Yamada [13], Hsu [14], and Ong [15] in East Asian populations, Vasilopoulos [16] in a Greek population, however, no significant findings by Mattei [17] in a Puerto Rican population, Grallert [18] and Niculescu [19] in European populations. These inconsistencies might be due to ethnicity, sample size and/or study design. In order to systematically evaluate the associations between *APOA5* gene *−1131T>C* polymorphism and fasting lipid parameters and the risk of metabolic syndrome, we conducted a case-control study in a Chinese population and a meta-analysis based on currently reported studies.

## Materials and Methods

### The case-control study

#### Study population and subjects

The case-control study was conducted from 2010–2011. Our study population was unrelated individual residents from Xiaoshan area, Zhejiang, P.R. China. A total of 905 MetS cases and 935 controls were recruited based on the following criteria. MetS was diagnosed according to the criteria of International Diabetes Federation (IDF) [20]. The recruitment criteria for cases were: all the subjects had central obesity (waist circumference (WC) ≥90 cm for males or ≥80 cm for females in Chinese) and at least met two of the following four criteria: (1) high TG level (≥150 mg/dl or 1.7 mmol/L), or specific treatment for this lipid abnormality; (2) low HDL-C (< 40 mg/dl or 1.03 mmol/L in males and < 50 mg/dl or 1.29 mmol/L in females), or specific treatment for this lipid abnormality; (3) high blood pressure (BP) (systolic BP≥130 or diastolic BP≥85 mmHg), or treatment of previously diagnosed hypertension; (4) high fasting plasma glucose (FPG) (FPG≥100 mg/dl or 5.6 mmol/L), or previously diagnosed type 2 diabetes mellitus (T2DM). The recruitment criteria for controls are the subjects with no history of obesity, hyperlipidaemia, dyslipidaemia, hypertension or diabetes mellitus.

#### Ethics statement

All participants were given and signed the written informed consent form and the study protocol was approved by the Institutional Review Board of School of Public Health, Zhejiang University.

#### Physical examination and lipid profiles

Height, weight and WC were measured while the subjects were dressed only in their undergarment and did not wear shoes after an overnight fast. At the same time, 2 ml of peripheral venous blood was drawn from each subject. Body mass index (BMI) was calculated as weight in kilograms divided by the square of height in meters (kg/m^2^). Systolic blood pressure (SBP) and diastolic blood pressure (DBP) was measured while subjects were in the sitting position after 15 minutes of rest. The concentrations of fasting total cholesterol (TC), TG, LDL-C, and HDL-C in the plasma were analyzed by enzymatic methods with commercially available kits on a Hitachi 7180 Autoanalyzer (Hitachi Ltd, Tokyo, Japan). Glucose was analyzed by a glucose oxidase method with the Beckman Glucose Analyzer (Beckman Instruments, Irvine, CA, USA).

#### Genotype imputation of the *−1131T>C* (rs662799)

An ongoing GWAS project on MetS has been performed for all the subjects with Illumina Beadchip OmniExpress (Illumina, San Diego, CA, USA). The genotype of the *+1891T>C* (rs2266788) was included in this database. The *−1131T>C* was found to be in strong linkage disequilibrium with the *+1891T>C* (rs2266788) in East Asians (CHB plus JPT) from HapMap phase II database (D′  =  1,r∧2  =  0.838). Therefore, the genotype information of the *−1131T>C* was imputed using MACH 1.0 [21] with CHB plus JPT samples in HapMap phase II as reference. The genotype with quality score (R^2^-hat)  =  0.8488 was considered well imputed.

#### Statistical analysis

Continuous variables were expressed as mean ± standard deviation (SD). Differences between case and control groups were estimated by two independent variable t-test for continuous variables and by Chi-square test for categorical variables. The departure from Hardy–Weinberg equilibrium was computed by the goodness-of-fit *x* ^2^ test with PLINK software (version 1.0.6) [22]. The general linear model was performed to compare lipids levels among *APOA5* genotypes with age, gender as covariates.Risk of MetS was evaluated under the unconditional logistic regression model and the genotype under assumptions of additive and dominant genetic models, respectively. Statistical power was calculated by Quanto version 1.2.4 (http://hydra.usc.edu/gxe/). For disease trait, power was calculated on the basis of cases sample, a ratio of controls vs. cases, allelic frequency, genetic effect under an inheritance model, a significance level, and the disease prevalence in population level. For quantitative trait, power was calculated on the basis of sample size, allelic frequency, population mean, standard deviation, and R squared (R_G_). A *P* value ≤0.05 was considered to be statistically significant. All statistical analyses were performed using SPSS 16.0 (SPSS Inc., Chicago, IL, USA).

### Meta-analysis

#### Search strategy and selection

We conducted a comprehensive search of Pubmed, HugeNavigator, Medline and the Chinese National Knowledge Infrastructure Database (CNKI), using the following keywords: “*APOA5*”, or “apolipoprotein A5”, in combination with “polymorphism”, or “variant”, or “SNP”, or “mutation” without language restriction. The last search was updated in March 2012. These searches were supplemented by scanning reference lists and correspondence with authors.

#### Inclusion and exclusion criteria and data extraction

The inclusion criteria for studies were as follows: (1) the participants were greater than 18 years old; (2) the *−1131T>C* variant of *APOA5* gene was studied; (3) the indices of outcomes were fasting TC or TG or LDL-C or HDL-C, or MetS; (4) if there were multiple studies from the same population, only the paper with the largest population would be extracted. Animal studies, reviews, case reports and simply commentaries were excluded.

Two investigators (Chunxiao Xu and Rongpan Bai) extracted data independently. The discrepancies were resolved by discussion with a third author (Dandan Zhang). The following data were collected from each study: first author's name, year of publication, ethnicity, study population, sex, health condition, sample size, genotype and allele distributions among participants with and without MetS, and mean with standard deviation (SD) or standard error by genotypes.

#### Statistical analysis

The associations between the *−1131T>C* and MetS or lipid parameters were analyzed with a dominant model. For lipid parameters, weighted mean difference (WMD) and its 95% confidence interval (CI) were calculated. For dichotomous data on disease status, pooled odds ratio (OR) and 95% CI were calculated in overall and subgroup populations. The heterogeneity among the studies was assessed by the Chi square-test based Q-statistic and *I^2^* test. A significant level, *α*, was set as 0.1 because Q-statistic is considered underpowered. *P* value greater than 0.1 for the Q-statistic implies a lack of heterogeneity among studies. On this condition, the fixed-effect model (the Mantel–Haenszel method) [23] was adopted. Otherwise the random-effects model (the DerSimonian and Laird method) [24] would be used. To ensure a more conservative evaluation for the association between *APOA5 −1131T>C* polymorphism and lipid levels, random-effect model was employed. Hardy-Weinberg equilibrium (HWE) in each group was estimated by the Chi-square test. Studies deviating from HWE were not excluded because there was no benefit in excluding these studies from genetic meta-analysis [25]. Stratified analysis was performed according to the characteristics of participants.

We also conducted subgroup analysis by excluding studies deviating from HWE. Galbraith plot was used to detect potential sources of heterogeneity. As for publication bias, Eegg's linear regression and Begg's funnel plots were performed. And Duval and Tweedie's trim and fill method [26] was employed here to adjust for publication bias when the funnel plot showed asymmetry. Stata version 11 (Stata Corp, College Station, Texas, USA) was used for the statistical analysis. Statistical significance was defined as two-sided *P* value less than 0.05.

## Results

### The case-control study

The basic characteristics of the subjects between 905 MetS cases and 935 controls are summarized in Table 1. The mean age in case group was 59.9±10.8 years and 55.3±11.9 years in control group (*P* < 0.001). No statistical difference in gender was found between cases and controls (*P* > 0.05). BMI, WC, SBP, DBP, TC, TG, LDL-C, and fasting plasma glucose in case group were significantly higher, whereas HDL-C was significantly lower than those in control group (all *P*<0.001).

**Table 1:** Basic characteristics of participants in a case-control study of the effects of APOA5 *−1131T>C* on fasting plasma lipids and metabolic syndrome (MetS) risk in a Chinese population, 2010–2011.

Variables	Case (n = 905)	Control (n = 935)	Total (N = 1,840)
Age(year)	59.9±10.8*	55.3±11.9	57.5±11.6
Sex			
Man	477 (52.7)	492 (52.6)	969 (52.7)
Woman	428 (47.3)	443 (47.4)	871 (47.3)
Systolic blood pressure (mmHg)	157.8±17.1*	120.0±11.2	138.6±23.8
Diastolic blood pressure (mmHg)	92.1±10.5*	72.6±8.4	82.2±13.6
Total cholesterol (mmol/L)	5.1±1.0*	4.4±0.8	4.7±0.9
Triglycerides (mmol/L)	3.1±2.2*	1.1±0.3	2.1±1.9
LDL-cholesterol (mmol/L)	2.2±0.8*	2.0±0.6	2.1±0.7
HDL-cholesterol (mmol/L)	1.4±0.4*	1.7±0.4	1.5±0.4
Fasting plasma glucose (mmol/L)	6.2±2.0*	4.9±0.4	5.5±1.6
BMI (Kg/m^2^)	27.0±2.7*	21.5±1.9	24.2±3.6
Waist circumference, cm			
Man	92.6±7.3*	77.3±6.7	84.9±10.4
Woman	87.7±8.2*	74.0±6.7	80.8±10.2

All continuous variables are expressed in means±standard deviation (SD) and categorical variables are expressed in number with percentage in parentheses.

Abbreviations: LDL, low-density lipoprotein, HDL, high-density lipoprotein, BMI, body mass index.

* *P*<0.001 when comparing with controls.

Genotype distributions and allele frequencies of the *−1131T>C* between case and control groups are shown in Table 2. The genotype frequencies in control group were in HWE (*P* = 0.139). The frequency of the *−1131C* allele in MetS cases was significantly higher than that in controls (24.2% vs. 19.1%; *P* = 0.0002). Under the dominant model and adjusted for age and gender, the carriers with the *−1131C* allele were found to have an increased risk for MetS (OR  =  1.40, 95% CI =  1.15, 1.69). Additionally, significantly increased risk of MetS was also found under the additive model, with per-C allele OR of 1.32 (1.08–1.62).

**Table 2:** Genotype and allele distributions of the *−1131T>C* between 905 cases of MetS and 935 controls and their association with MetS risk in a case-control study in a Chinese population, 2010–2011.

Genotype/allele	Case	Control	*P* †	Additive model		Dominant model	
				OR (95% CI)‡	*P* ‡	OR (95% CI)‡	*P* ‡
*TT*	534 (59.0)	618 (66.1)		1.00	0.001	1.00	0.001
*TC*	304 (33.6)	276 (29.5)	0.001	1.32 (1.08–1.62)		1.40 (1.15–1.69)	
*CC*	67 (7.4)	41 (4.4)		1.88 (1.24–2.83)			
*C* allele (%)	24.2	19.1	0.0002				

† *P* value was calculated by the *x* ^2^ test.

‡ OR (odds ratio), 95% CI (confidence interval) and *P*-value were adjusted for age and sex.

Abbreviation: MetS, metabolic syndrome.

Table 3 presents lipid profiles of MetS cases, controls and the combined population according to the *– 1131T>C* genotype. Among the combined overall population, the C allele was significantly associated with increased plasma concentrations of TG (*P* < 0.001) and TC (*P* = 0.024), and decreased level of HDL-C (*P* = 0.007). In case group, the *−1131C* allele was also related to higher TC, TG, LDL-C and lower HDL-C while significance was only observed for TG (*P* = 0.001). In control group, although the associations between the *−1131T>C* and lipids were not statistically significant, the trends were consistent.

**Table 3:** Lipid profiles of MetS cases, controls, and combined population according to APOA5 *−1131T>C* genotype in a case-control study in a Chinese population, 2010–2011.

	Case			Control			Total		
Parameters	*TT*	*TC/CC*	*P* ¶	*TT*	*TC/CC*	*P* ¶	*TT*	*TC/CC*	*P* ¶
N (%)	534 (62.7)	371 (37.3)		618 (66.1)	317 (33.9)		1152 (62.6)	688 (37.4)	
Total cholesterol (mmol/L)	5.0±0.9	5.1±0.9	0.475	4.3±0.8	4.4±0.8	0.296	4.7±0.9	4.8±0.9	**0.024**
Triglycerides (mmol/L)	2.9±2.0	3.4±2.1	**0.001**	1.1±0.3	1.2±0.3	0.389	2.0±1.6	2.3±1.9	**<0.001**
LDL-cholesterol (mmol/L)	2.2±0.7	2.3±0.8	0.056	1.9±0.6	2.0±0.6	0.051	2.1±0.7	2.1±0.7	0.752
HDL-cholesterol (mmol/L)	1.5±0.4	1.4±0.4	0.193	1.7±0.4	1.6±0.4	0.162	1.6±0.4	1.5±0.4	**0.007**

¶ *P* value was tested by the general linear model, adjusted for age and sex.

Abbreviations: MetS, metabolic syndrome; LDL, low-density lipoprotein; HDL, high-density lipoprotein.

### The meta-analysis

Fifty-eight eligible papers were extracted and our own case-control study was included for meta-analysis (Figure S1). Ninty-one studies with a total of 51,868 subjects were included in the analysis of *APOA5* gene-lipid associations [7]–[19], [27]–[71] (Table S1). Among these studies, 26 studies were of European origin, 46 East Asian origin (predominantly China and Japan), and 19 in other regions or multinational. A total of 63, 82, 54 and 69 studies were included for comparing the difference in plasma TC, TG, LDL-C and HDL-C, respectively (Tables S1, S2). Twelve studies with 5,573 cases and 8,290 controls were included in the analysis of *APOA5* gene-MetS association [13]–[19], [30], [51], [63] (Table S3). Among them, 5 studies were of European, 5 East Asian, and 2 other ethnicity (Puerto Rican descent and Turkish, respectively).

#### The *−1131T>C* polymorphism and fasting lipid levels

The *−1131C* carriers had an increased TC by 0.08 mmol/L (95% CI  =  0.05, 0.10; *P* = 1.74×10^−9^) compared with the TT group (Table 4). Subgroup analysis stratified by the characteristics of the subjects was further conducted, as presented in Table S4. Higher TC level in the *−1131C* carriers than non-carriers was also observed in the subgroups but not statistically significant in MetS, coronary heart disease (CHD), man, and woman subgroups.

**Table 4:** Meta-analysis of the effects of *APOA5 −1131T>C* on fasting plasma lipids and MetS risk.

Groups	Studies (n)	*I^2^* (%)	Q test *P* value	WMD (95% CI)	*P*
**TC**					
All	63	0.0	0.512	0.08 (0.05, 0.10)	1.74×10^−9^
All in HWE	60	2.8	0.413	0.08 (0.05, 0.11)	1.35×10^−8^
**TG**					
All	84	68.0	0.00001	0.30 (0.26, 0.33)	1.87×10^−55^
All in HWE	82	68.7	0.00001	0.30 (0.26, 0.34)	2.85×10^−53^
**LDL-C**					
All	54	19.7	0.109	0.04 (0.02, 0.07)	0.002
All in HWE	51	21.3	0.095	0.04 (0.01, 0.07)	0.006
**HDL-C**					
All	69	39.6	0.001	−0.05 (−0.06, −0.04)	1.88×10^−21^
All in HWE	66	38.0	0.001	−0.05 (−0.06, −0.04)	5.94×10^−20^
**MetS**					
All	12	55.4	0.010	1.33 (1.16, 1.53)	0.00004
All in HWE	11	50.2	0.029	1.30 (1.14, 1.47)	0.00007

Abbreviations: WMD, weighted mean difference; HWE, Hardy-Weinberg Equilibrium; MetS, metabolism syndrome.

Mean plasma TG levels for *−1131C* carriers increased 0.30 mmol/L (95% CI  =  0.26, 0.33; *P* = 1.87×10^−55^) compared with the TT group (Table 4). And the similar results were also observed in all subgroups (Table S4).

Mean plasma LDL-C levels for *−1131C* carriers increased 0.04 mmol/L (95% CI = 0.02, 0.07; *P* = 0.002) compared with the TT group (Table 4). Weak but significant association between this variant and higher LDL-C was detected in East Asians, studies with large samples and studies with small samples. The effect trends in the other subgroups such as Europeans, Man, CHD, Type 2 diabetes subjects also showed consistent but no significance was observed (Table S4).

Mean plasma HDL-C levels in the CC+TC group were decreased, on the average, to 0.05 mmol/L (95% CI  =  −0.06, −0.04; *P* = 1.88×10^−21^) compared with the TT group (Table 4). The subgroup analysis showed a significant association except for man and CHD patients while the direction of effect in the two subgroups was consistent (Table S4).

Moreover, further analysis excluding 3 studies not in HWE was also performed. The overall and subgroup analysis results were similar to those when the studies not in HWE were included.

#### The *−1131T>C* genotypes and metabolic syndrome

**Figure 1:**
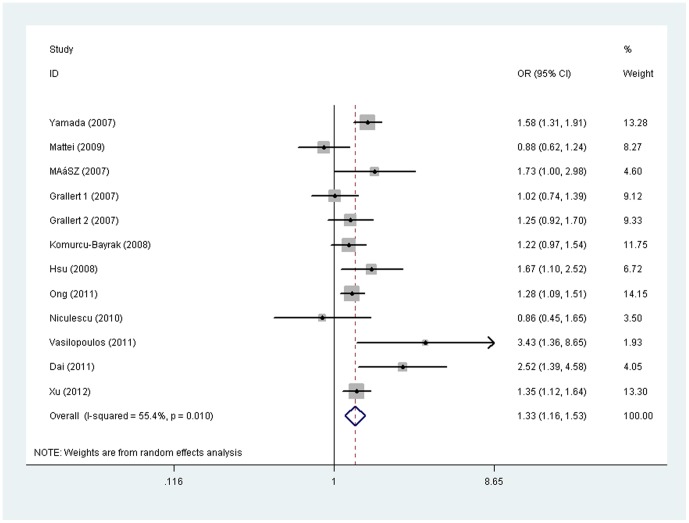
Forest plot of odds ratio (OR) and 95% confidence interval (CI) for each study of the association between the *−1131T>C* polymorphism and metabolic syndrome risk under dominant model (carriers vs. non-carriers) in meta-analysis. The squares and horizontal lines correspond to OR and 95% CI for each study, and the area of squares reflects study weight (inverse of the variance). The diamond represents the pooled OR and its 95% CI.

A significant association was observed between the *−1131T>C* polymorphism and MetS under dominant model (CC+TC vs. TT): pooled OR =  1.33, 95% CI  =  1.16, 1.53, *P* =  0.00004 (Table 4). Similar results were found after excluding a study not in HWE. Forest plot on the basis of all available studies is shown in Figure 1. In the subgroup analysis by ethnicity of study population, significant association between the C allele and MetS risk was only detected in East Asians: pooled OR  =  1.43, 95% CI  =  1.29, 1.58, *P* =  2.5×10^−12^. However, this significance was not detected in European populations, pooled OR (95% CI): 1.30 (0.94, 1.78). When stratifying by study design, significant association was detected in both population-based (pooled OR  =  1.23, 95% CI = 1.13, 1.35) and hospital-based studies (pooled OR  =  1.70, 95% CI  =  1.44, 2.01) (Table S4).

### Heterogeneity

Significant heterogeneity was observed in the studies on TG, HDL-C and MetS (Table 4). By using Galbraith plot, 13, 6, 3 studies were identified as main contributors to heterogeneity on TG, HDL-C and MetS, respectively, (Figures S4, S5, and S6). After excluding the outlier studies, the heterogeneity was effectively disappeared or decreased. Pooled WMDs and ORs were recalculated after removal of the outlier studies, and the pooled results were similar to those when the outlier studies were included with the exception of the association between the *−1131C* allele and HDL-C in CHD patients (Tables S5).

### Publication bias

With Begg's funnel plot and Eegg's test, no publication bias was detected among the studies on the association of the *−1131T>C* polymorphism and TC, LDL-C, HDL-C, or MetS risk (Figures S2, S3). However, an asymmetry funnel plot detected for the *−1131T>C* and serum TG levels suggested the presence of publication bias. After adjustment with trim and fill method, the pooled WMD was reduced to 0.18 (0.14, 0.21) compared with unadjusted pooled WMD, but this was still statistically significant indicating that the results were statistically robust.

## Discussion

In this study, we evaluated associations between the genetic variation of the *−1131T>C* of *APOA5* gene and lipid levels and the risk for MetS in a case-control study in a Chinese population and meta-analysis. In the case-control study, the individuals with the *−1131C* allele had a higher level of fasting TG in MetS cases, higher TC, TG and lower HDL-C and 40% increased MetS risk in the overall Chinese population. The meta-analysis of gene-lipid association in 51,868 participants showed that the *−1131C* allele was associated with higher levels of fasting TG, TC, LDL-C and lower level of HDL-C. Moreover, the gene-MetS association meta-analysis pooling data from 11 previous studies and the current containing 13,863 (5,573 cases and 8,290 controls) subjects also confirmed that individuals with the *−1131C* allele had a 33% increased risk to develop MetS.

Polymorphisms in or near the *APOA5* gene are among the strongest known genetic determinants of plasma TG concentration, and also have been reported to be associated with higher TC, LDL-C and lower HDL-C concentration [11]–[12], [72]. Study of variations in *APOA5* gene can help to clarify the relevance of the *APOA5* pathway to lipid metabolism and MetS risk. The *−1131T>C* located in the promoter region of *APOA5* gene, might down-regulate *APOA5* mRNA translation, which in turn results in lower apo A5 levels, and then leads to higher levels of TG [73]. The mechanism governing the contribution of the *−1131T>C* in the reduction of gene expression remains to be studied. The *−1131T>C* has been observed in modest to strong linkage disequilibrium with another locus, *+1891T>C*, in different populations [59], [62]. The *+1891T>C* was associated with MetS in a European GWAS conducted by STAMPEED Consortium [3] whereas the *−1131T>C* and *+1891T>C* were not in the same haplotype block in their study population. Till now, few variants in or near this gene have been reported associated with MetS by relative few European GWASs. Another intergenic locus in *APOA1/C3/A4/A5* gene cluster region, rs964184, was identified associated with MetS in Finnish populations by a GWAS [4].

The genotype of the *−1131T>C* was imputed according to directly genotyped *+1891T>C* in our GWAS data. With a strong disequilibrium, the observed genotypes provide adequate information about the unobserved genotypes, and the imputation algorithm can infer genotypes with high confidence [74]. The *−1131T>C* was found to be in strong linkage disequilibrium with the *+1891T>C* in East Asians from HapMap database. And the genotyping quality score (R^2^-hat)  =  0.8488 suggested that the *−1131T>C* was well imputed.

Meta-analysis in 11,583 European individuals showed that the −*1131T>C* was related to TC, TG and HDL-C, however, unrelated to LDL-C. These findings are similar to a previous meta-analysis mainly consisting of Europeans conducted by Triglyceride Coronary Disease Genetics Consortium and Emerging Risk Factors Collaboration, in which they found that for every C allele of the *APOA5 –1131T>C* genotype inherited, mean TG level was higher and mean HDL-C level was lower [75]. Another meta-analysis by Saleheen *et al.* [76] was conducted in merely Europeans in which they only explored the effect size of the *−1131T>C* on TG and also found a positive association.

To our knowledge, this is the first study to conclude a positive association between the *−1131C* and LDL-C in East Asians. A total of 24,563 East Asian individuals were included in the meta-analysis which ensured adequate power. Mean plasma LDL-C level for the *−1131C* carriers was 0.04 mmol/L (0.02, 0.07) higher than that for the TT carriers (*P* = 0.001). There are some possibilities for the discrepancy between Europeans and East Asians. First, genotypic effects could be ethnicity specific. Compared with Europeans (7.7% in the meta-analysis), the minor allele frequency of the *−1131T>C* among East Asians was much higher (28.6% in the meta-analysis). Second, there are different lifestyle environmental factors such as diet especially animal food consumption and alcohol use which have been reported to play a role in modifying the effect of *APOA5* on plasma lipids [17], [54]. Besides, some uncovered additional gene-gene interactions may occur in different populations.

MetS in our case-control study was defined by IDF while most of our available studies were defined by NCEP ATP III [77]. However, meta regression analysis indicated that the definition of MetS did not contribute to heterogeneity. A previous meta-analysis by Povel *et al.* [78] concluded that the *−1131C* allele was associated with increased MetS risk in overall population and in Europeans. Our larger sample with 5,573 cases and 8,290 controls with 5 studies in East Asians and 5 studies in Europeans and 2 in other ethnic studies provided higher OR: 1.33 (1.16, 1.53) and the novel finding that positive association was only detected in East Asians but not in Europeans. To our knowledge, it was the first meta-analysis to provide significant evidence for this association in East Asian populations. We noted that a study in Europeans by Dallongeville [79] in which actually another locus (*ser19trp, C56G*) was reported to have a positive association with MetS was included in the Povel's. This study may be the source of the inconsistent conclusions in Europeans between our meta-analysis and the Povel's. In our study, inconsistency between Europeans and East Asians can be explained by diverse gene-environment interactions in different ethnic groups [80]. And different genotype and/or allele frequencies of this locus in different populations may predispose people to various degrees of MetS susceptibility [81]. Relative small sample size in Europeans might cause the inconspicuousness also.

Heterogeneity was detected among studies comparing the levels of TG and HDL-C and risk of MetS between the *−1131C* allele carriers and non-carriers. With subgroup analysis, heterogeneity was removed in some subgroups but still existed in others. By using Galbraith plot, outlier studies contributing to heterogeneity were identified. After exclusion of these outlier studies, heterogeneity was effectively disappeared or decreased while the recalculated results remained similar with the exception of the association between the *−1131C* allele and HDL-C in CHD patients. This indicated that more studies should be conducted to further examine the association of this polymorphism with HDL-C in CHD patients.

Publication bias was only observed in studies on the association between the *−1131T>C* polymorphism and plasma TG concentration. It is known that the existence of publication bias may change the conclusion of a meta-analysis. Here using Duval and Tweedie's trim and fill method, a pooled WMD adjusted for publication bias was calculated. And the result with slightly lower mean TG level remained significant suggesting the reliability of the whole pooled result.

There are several major strengths in our study. First, our study confirmed and extended the previous findings that the *APOA5 −1131C* allele was associated with elevated levels of fasting TG, TC, LDL-C and decreased HDL-C, and associated with increased risk of MetS, especially in East Asians. Second, it was the first meta-analysis to provide significant evidence for the associations of the *APOA5 −1131T>C* with LDL-C and MetS in East Asian populations. Third, our study adds additional evidence of the effect size of the *−1131T>C* on lipid levels in East Asians which has not been investigated either by Triglyceride Coronary Disease Genetics Consortium and Emerging Risk Factors Collaboration or by Saleheen. Fourth, the statistical power of both our case-control study and meta-analysis is adequate (94.9% for TG and 94.4% for MetS in case-control study and 100% for meta-analysis) which supports the reliability of the results.

Meanwhile, there are potential limitations in this study. First, although we only explored the effect of *APOA5 −1131T>C* variant on plasma lipid levels and the prevalence of metabolic syndrome, there are still many other *APOA5* SNPs, gene-gene and/or gene-environment interactive factors needed to be considered. For example, polymorphisms in the *APOC3* gene have been shown to interact with *APOA5* gene and affect plasma TG concentration [29]. Lifestyles including diet pattern, smoking, alcohol use, physical activities are important factors for lipid regulation and prevalence of MetS. Either in our own study or in the meta-analysis, however, such information is unavailable. Second, this study has not looked into the haplotype of *APOA5* gene which may be related to our studied outcomes. Third, the genotype of the *−1131T>C* was imputed instead of directly genotyped. Therefore we make a cautious conclusion.

In conclusion, the *APOA5 −1131C* allele may be associated with increased levels of fasting TG, TC, LDL-C and decreased HDL-C, and associated with increased risk of MetS, especially in East Asians.

## Supporting Information

Figure S1 Flow chart of study identification.
(TIF)

Figure S2 Funnel plot for *−1131T>C* and TC, TG, LDL-C and HDL-C under dominant model (CC/CT vs. TT). Each point represents a separate study for the indicated association. SE (WMD), standard error (weighted mean difference). (A) The funnel plot comparing the differences in TC; (B) The funnel plot comparing the differences in TG; (C) The funnel plot comparing the differences in LDL-C; (D) The funnel plot comparing the differences in HDL-C.
(TIF)

Figure S3 Funnel plot for *−1131T>C* and metabolic syndrome under dominant model (CC/CT vs. TT). Each point represents a separate study for the indicated association. SE (logor), standard error of log (odds ratio).
(TIF)

Figure S4 Galbraith plot of *APOA5 −1131T>C* polymorphism and plasma TG under dominant model (CC/CT vs. TT).
(TIF)

Figure S5 Galbraith plot of *APOA5 −1131T>C* polymorphism and plasma HDL-C under dominant model (CC/CT vs. TT).
(TIF)

Figure S6 Galbraith plot of *APOA5 −1131T>C* polymorphism and risk of metabolic syndrome under dominant model (CC/CT vs. TT).
(TIF)

Table S1 Characteristics of the individual included studies with fasting lipid levels as outcome.
(DOC)

Table S2 Plasma lipid levels by genotypes of individual studies included in the meta-analysis for the *−1131T>C*.
(DOC)

Table S3 Studies included in the meta-analysis of the association of *APOA5 −1131T>C* with metabolic syndrome.
(DOC)

Table S4 Subgroup analysis in a meta-analysis of the effects of *APOA5 −1131T>C* on fasting plasma lipids and metabolic syndrome risk.
(DOC)

Table S5 Meta-analysis of the effects of *APOA5 −1131T>C* on fasting plasma lipids and metabolic syndrome risk (excluding the outlier studies).
(DOC)
