# Supplementary figures and images for: Effects of APOA5 −1131T>C (rs662799) on Fasting Plasma Lipids and Risk of Metabolic Syndrome: Evidence from a Case-Control Study in China and a Meta-Analysis
Source: PLoS One. 2013 Feb 28;8(2):e56216. doi: 10.1371/journal.pone.0056216 (PMC3585417; doi:10.1371/journal.pone.0056216)

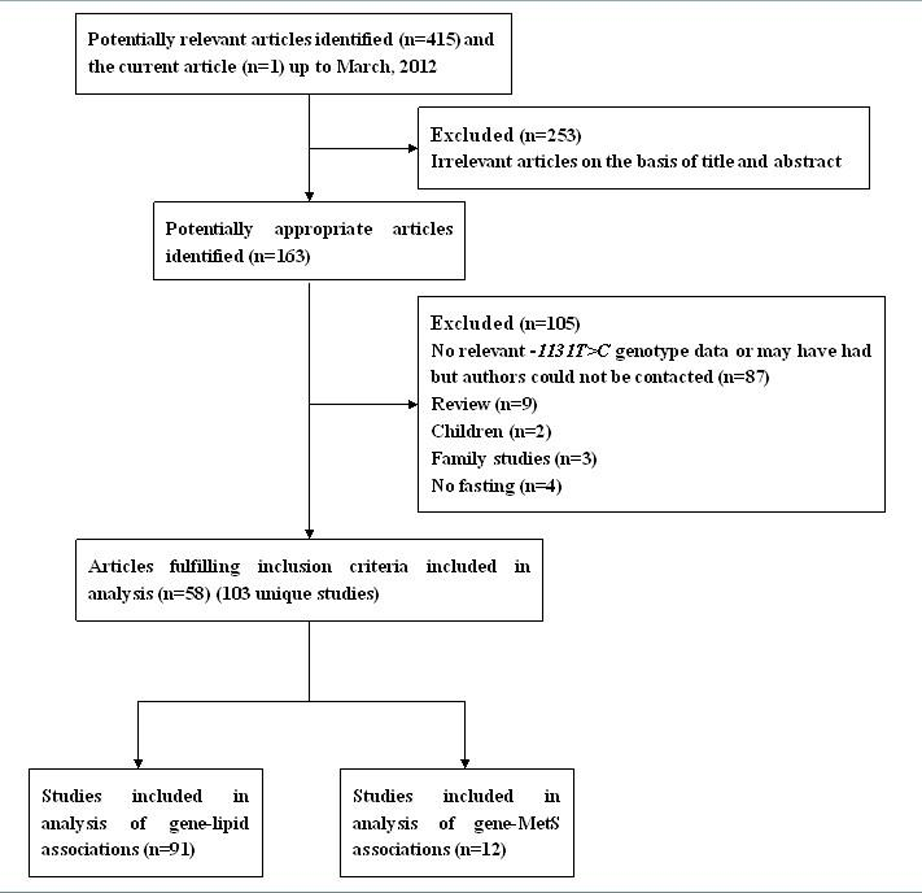

Supplement: Figure S1 — Flow chart of study identification. (TIF) [file pone.0056216.s001.tif]

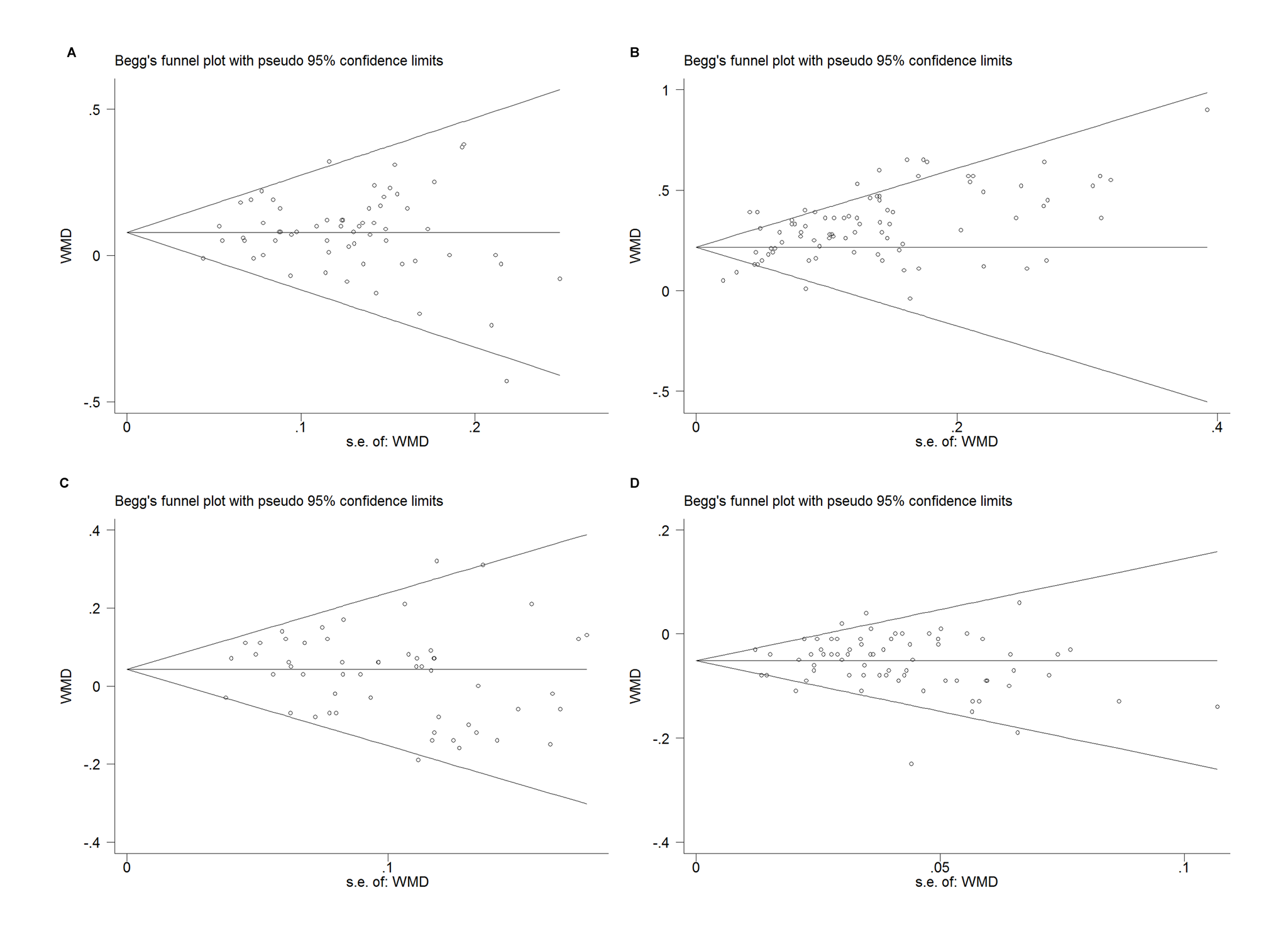

Supplement: Figure S2 — Funnel plot for −1131T>C and TC, TG, LDL-C and HDL-C under dominant model (CC/CT vs. TT). Each point represents a separate study for the indicated association. SE (WMD), standard error (weighted mean difference). (A) The funnel plot comparing the differences in TC; (B) The funnel plot comparing the differences in TG; (C) The funnel plot comparing the differences in LDL-C; (D) The funnel plot comparing the differences in HDL-C. (TIF) [file pone.0056216.s002.tif]

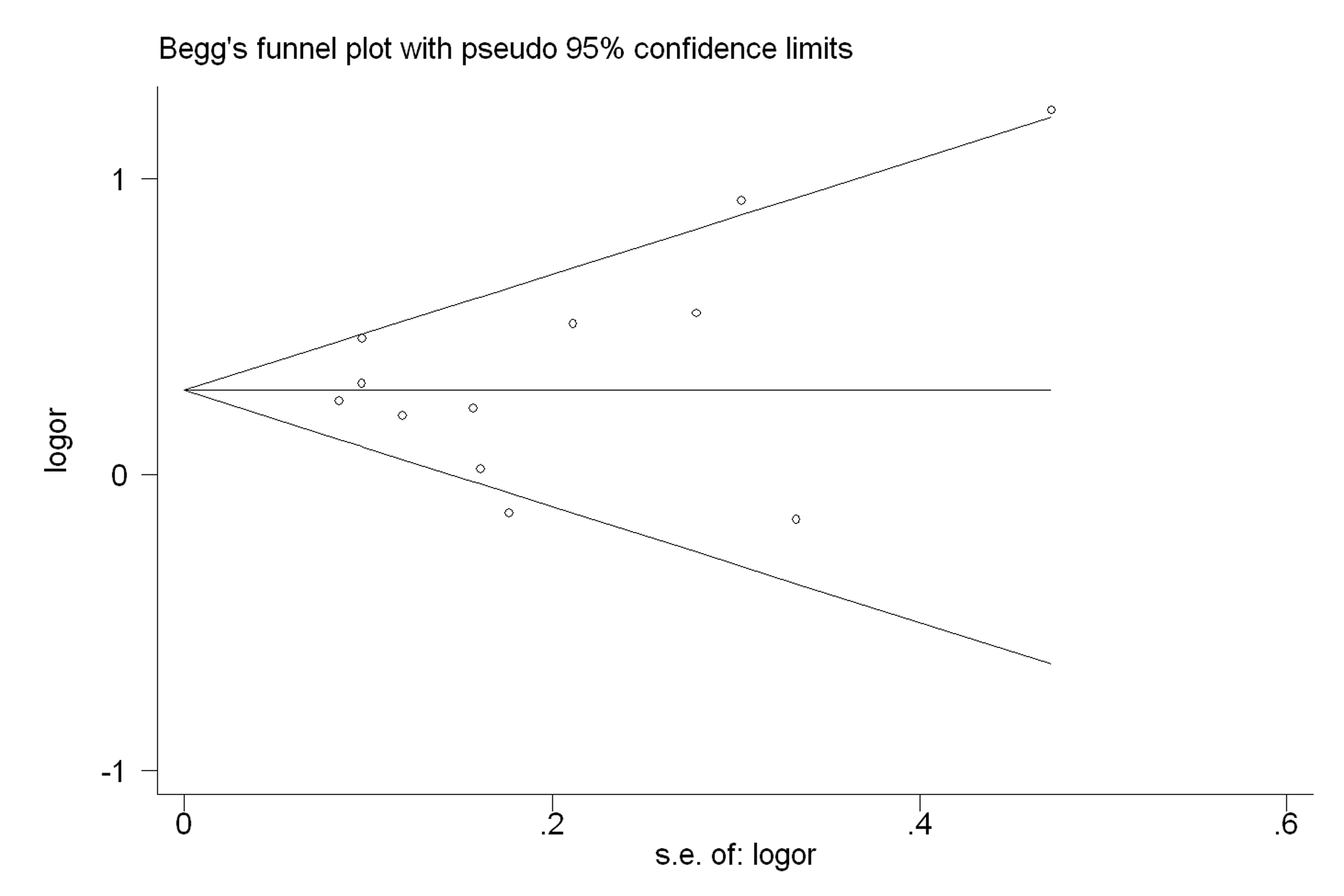

Supplement: Figure S3 — Funnel plot for −1131T>C and metabolic syndrome under dominant model (CC/CT vs. TT). Each point represents a separate study for the indicated association. SE (logor), standard error of log (odds ratio). (TIF) [file pone.0056216.s003.tif]

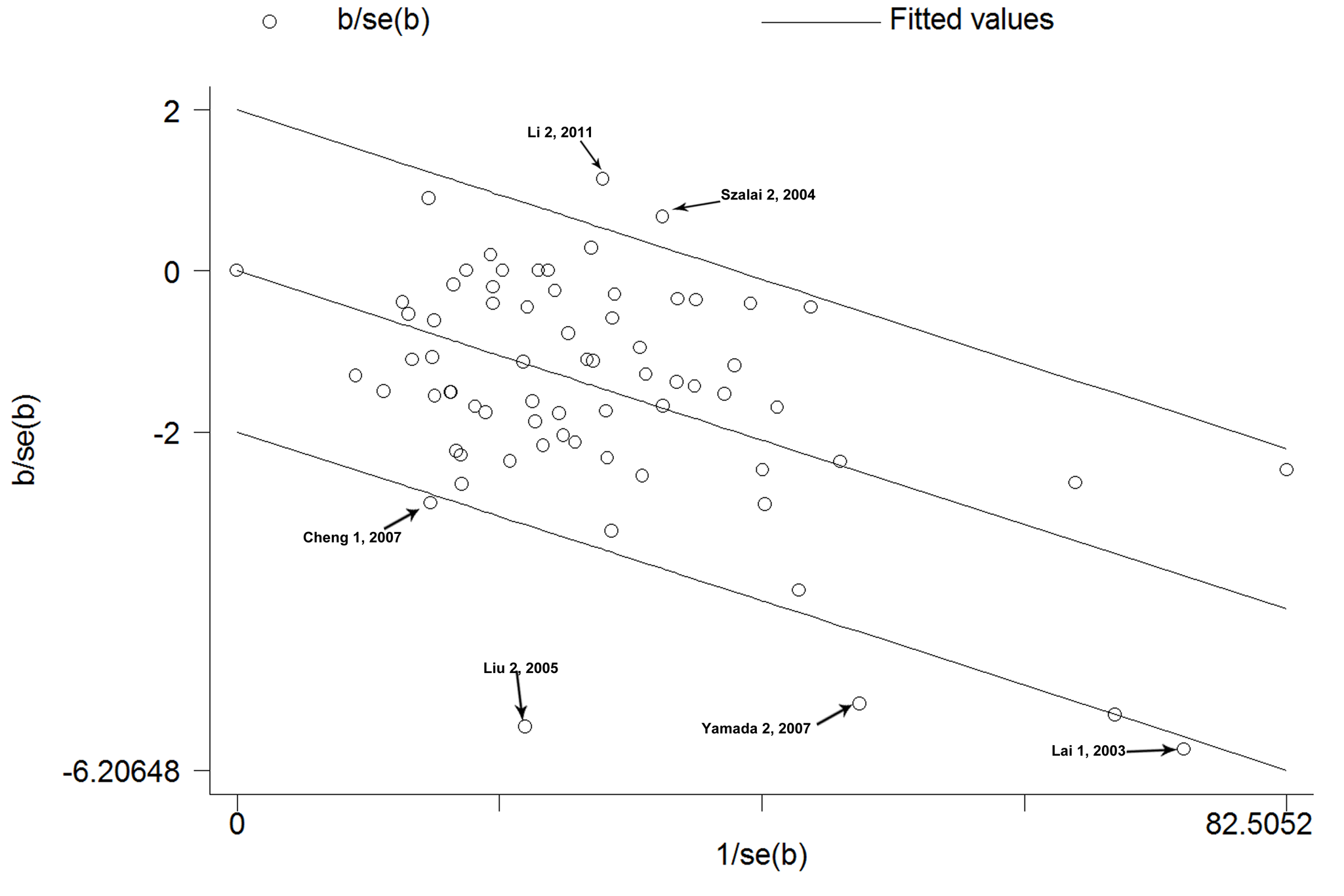

Supplement: Figure S5 — Galbraith plot of APOA5 −1131T>C polymorphism and plasma HDL-C under dominant model (CC/CT vs. TT). (TIF) [file pone.0056216.s005.tif]

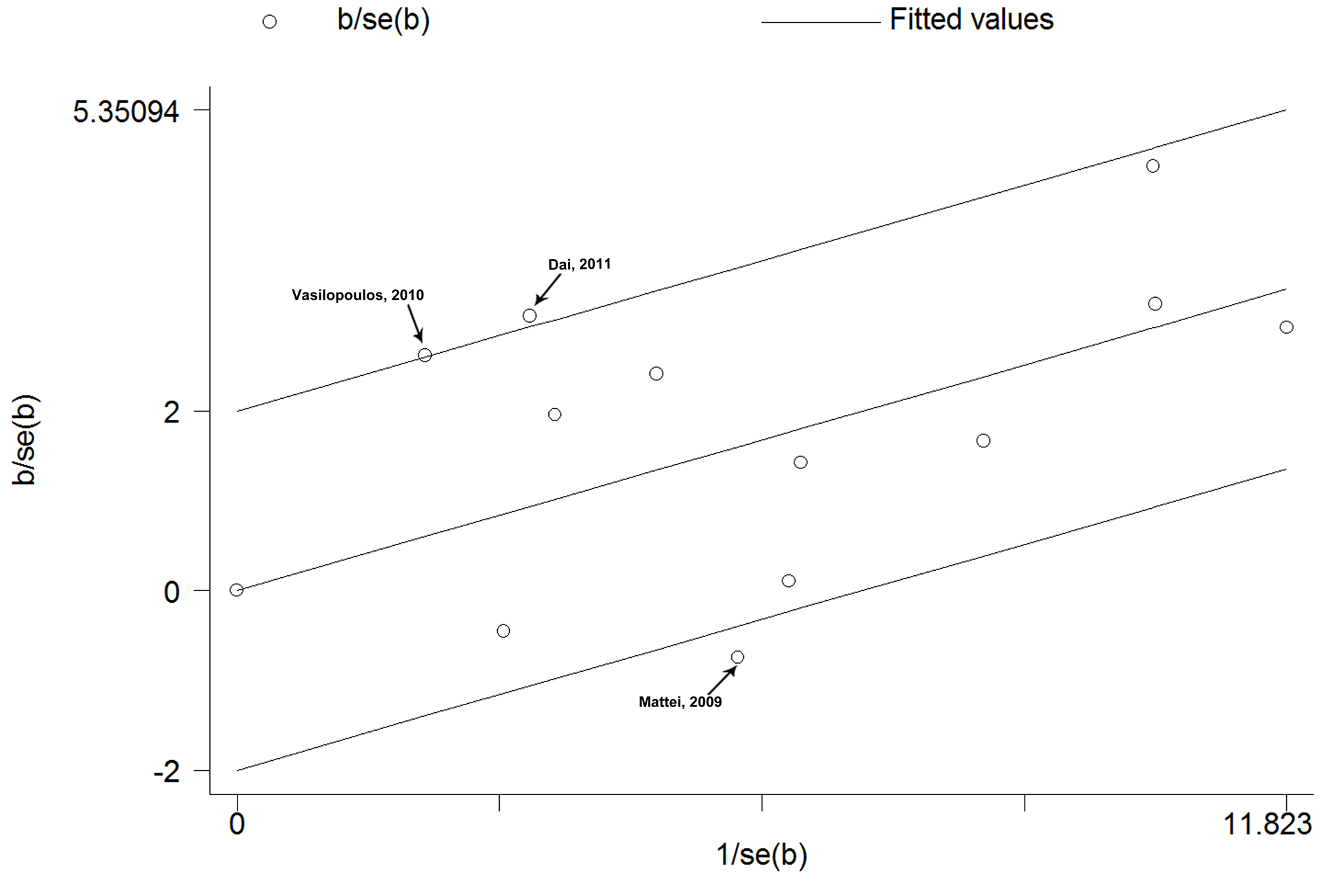

Supplement: Figure S6 — Galbraith plot of APOA5 −1131T>C polymorphism and risk of metabolic syndrome under dominant model (CC/CT vs. TT). (TIF) [file pone.0056216.s006.tif]
