# Supplementary material for: Effects of APOA5 −1131T>C (rs662799) on Fasting Plasma Lipids and Risk of Metabolic Syndrome: Evidence from a Case-Control Study in China and a Meta-Analysis
Source: PLoS One. 2013 Feb 28;8(2):e56216. doi: 10.1371/journal.pone.0056216 (PMC3585417; doi:10.1371/journal.pone.0056216)
Supplement: Table S1 — Characteristics of the individual included studies with fasting lipid levels as outcome. (DOC) [file pone.0056216.s007.doc]

**Table S1. Characteristics of the individual included studies with fasting lipid levels as outcome.**

| First author, year, reference | Ethnicity | Gender | Take lipid-lowering medication | Study population ( population source) | Outcomes |
| --- | --- | --- | --- | --- | --- |
| Lai 1, 2003 [12]  Lai 2, 2003 [12]  Lai 3, 2003 [12]  Chaaba 1, 2005 [27]  Chaaba 2, 2005 [27]  Járomi 1, 2010 [28]  Járomi 2, 2010 [28]  Chien, 2008 [29]  Maasz 1, 2007 [30]  Maasz 2, 2007 [30]  Maasz 1, 2008 [61]  Maasz 2, 2008 [61]  Charriere, 2008 [31]  Kisfali 1, 2010 [32]  Kisfali 2, 2010 [32]  Martinelli, 2007 [33]  Talmud, 2004 [34]  Hubacek 1, 2008^c^ [35]  Hubacek 2, 2008 [35]  Mattei, 2009 [17]  Hahne, 2008 [36]  Sundl, 2007 [37]  Elosua, 2006 [38]  Szalai 1, 2004 [39]  Szalai 2, 2004 [39]  Hodoglugil 1,2006 [40]  Hodoglugil 2, 2006 [40]  Hodoglugil 3, 2006 [40]  Hodoglugil 4, 2006 [40]  Pennacchio 1,2003 [6]  Pennacchio 2,2003 [6]  Pennacchio 3,2003 [6]  Pennacchio 4,2003 [6]  Pennacchio 5,2003 [6]  Pennacchio 6,2003 [6]  Huang, 2008 [7]  Girona, 2008 [41]  Jang 1, 2009 [42]  Jang 2, 2009 [42]  Yamada 1, 2007 [43]  Yamada 2, 2007 [43]  Li 1, 2008 [44]  Li 2, 2008 [44]  Li 3, 2008 [44]  Hsu, 2006 [45]  Bi 1, 2004 [46]  Bi 2, 2004 [46]  Baum, 2003 [8]  Hsu, 2008 [14]  Baum 1, 2007 [47]  Baum 2, 2007 [47]  Baum 3, 2007 [47]  Vasilopoulos, 2010 [16]  Olano-Martin,2008 [48]  Jang, 2004 [49]  Aouizerat, 2003 [9]  Li, 2004 [50]  Komurcu-Bayrak 1,  2008 [51]  Komurcu-Bayrak 2, 2008 [51]  Lee, 2004 [52]  Li 1, 2011 [53]  Li 2, 2011 [53]  Yamada 2, 2007 [13]  Yamada 1, 2007 [13]    Yin 1, 2011 [54]  Yin 2, 2011 [54]  Lin , 2011 [55]  Yan 1, 2005 [55]  Yan 2, 2005 [56]  Yan 3, 2005 [56]  Song, 2012 [57]  Chandak 1, 2006 [58]  Chandak 2, 2006 [58]  Jang, 2010 [10]  Ken-Dror 1, 2010 [59]  Ken-Dror 2, 2010 [59]  Ken-Dror 3, 2010 [59]  Qiao 1, 2008 [64]  Qiao 2, 2008 [64]  Zhu, 2007 [65]  Tang, 2005 [71]  Qiu, 2007 [66]  Wang, 2010 [67]  Li, 2007 [68]  Cheng 1, 2007 [69]  Cheng 2, 2007 [69]  Cheng 3, 2007 [69]  Yang, 2007 [70]  Liu 1, 2005 [62]  Liu 2, 2005 [62]  Xu 1, 2012  Xu 2, 2012 | East Asian  Other  Other  Other  Other  Caucasian  Caucasian  East Asian  Caucasian  Caucasian  Caucasian  Caucasian  Caucasian  Caucasian  Caucasian  Caucasian  Caucasian  Caucasian  Caucasian  Other  Caucasian  Caucasian  Caucasian  Caucasian  Caucasian  Caucasian  Caucasian  Other  Other  Caucasian  Caucasian  Other  Other  Other  Other  East Asian  Caucasian  East Asian  East Asian  East Asian  East Asian  East Asian  East Asian  East Asian  East Asian  East Asian  East Asian  East Asian  East Asian  East Asian  East Asian  East Asian  Caucasian  Other  East Asian  Other  East Asian  Other  Other  Caucasian  East Asian  East Asian  East Asian  East Asian  East Asian  East Asian  East Asian  East Asian  East Asian  East Asian  East Asian  Caucasian  Other  East Asian  Other  Other  Other  East Asian  East Asian  East Asian  East Asian  East Asian  East Asian  East Asian  East Asian  East Asian  East Asian  East Asian  East Asian  East Asian  East Asian  East Asian | M/F  M/F  M/F  M/F  M/F  M/F  M/F  M/F  M/F  M/F  M/F  M/F  M/F  M/F  M/F  M/F  M  M  F  M/F  M/F  M  M/F  M/F  M/F  M  F  M  F  F  M  F  M  F  M  F  M/F  M/F  M/F  M/F  M/F  M/F  M/F  M/F  M/F  M/F  M/F  M  M/F  M/F  M/F  M/F  M/F  M/F  M  M/F  M/F  F  M  M/F  M/F  M/F  M/F  M/F    M/F  M/F  M/F  M/F  M/F  M/F  M/F  M/F  M/F  M/F  M/F  M/F  M/F  M/F  M/F  M/F  M/F  M/F  M/F  M/F  M/F  M/F  M/F  M/F  M/F  M/F  M/F  M/F | NA  NA  NA  No  No  NA  NA  No  NA  NA  NA  NA  Partial use  NA  NA  No  NA  NA  NA  Partial use  Partial use  NA  NA  No  No  No  No  No  No  NA  NA  NA  NA  NA  NA  NA  NA  No  Partial use  NA  NA  NA  NA  NA  NA  No  No  NA  NA  No  Partial use  Partial use  No  No  No  Ceased lipid- lowering medication for at least 1 month  No  No  No  NA  NA  NA  No  NA  No  No  No  No  No  No  NA  NA  NA  No  Partial use  Partial use  Partial use  NA  NA  NA  No  NA  NA  NA  NA  NA  NA  Partial use  No  No  NA  NA | Random subjects (General)  Random subjects (General)  Random subjects (General)  Subjects with type 2 diabetes (Hospital/trial)  Subjects without type 2 diabetes  (Hospital/trial)  Ischemic stroke patients (General)  Control subjects with negative brain MRI findings (General)  Individuals without hypertriglyceridemia  (Hospital/trial)  Patients with metabolic syndrome (Hospital/trial)  Control subjects (General)  Patients with ischemic stroke  Individuals free from neuro-imaging alterations and clinical history of stroke  Type 2 diabetic patients (Hospital/trial)  Metabolic syndrome patients (General)  Healthy subjects (General)  Subjects with CAD and subjects without CAD (General)  Subjects with CHD (Hospital/trial)  MONICA study (General)  MONICA study (General)  Random older adults (General)  Obese patients and non-obese subjects (Hospital/trial)  Healthy nonsmoking subjects (General)  Framingham Offspring Study  (General)  Patients with severe CAD (Hospital/trial)  Subjects without CAD (Hospital/trial)  Random subjects from American Bank employees (General)  Random subjects from American Bank employees (General)  Random subjects from Turkish Heart Study (General)  Random subjects from Turkish Heart Study (General)  Random sample of Dallas County residents (General)  Random sample of Dallas County residents (General)  Random sample of Dallas County residents (General)  Random sample of Dallas County residents (General)  Random sample of Dallas County residents (General)  Random sample of Dallas County residents (General)  Hypertriglyceridemic and control subjects (General)  Non-smoker type 2 diabetic patients (Hospital/trial)  Healthy control subjects (General)  Coronary artery disease patients  (Hospital/trial)  Random subjects visiting hospital (Hospital/trial)  Community-dwelling elderly individuals (General)  Healthy subjects (Hospital/trial)  Type 2 diabetic patients (Hospital/trial)  Type 2 diabetic patients with cerebral infarction (Hospital/trial)  Subjects with MetS (Hospital/trial)  Patients with CHD (Hospital/trial)  Apparently healthy individuals (Hospital/trial)  Subjects with high or low triglyceride (Hospital/trial)  Subjects with and without metabolic syndrome (Hospital/trial)  Normal controls (Hospital/trial)  Type 2 diabetic patients without diabetic nephropathy (Hospital/trial)  Type 2 diabetes patients with diabetic nephropathy (Hospital/trial)  Subjects with MetS and controls  (Hospital/trial)  Healthy adults (General)  Healthy men (General)  Subjects with combined hyperlipidemia, subjects with hypoalphalipoproteinemia, subjects with hyper alphalipoproteinemia, and control subjects (General)  Volunteers without hyperlipoproteinemia, hypertension,diabetes,endocrineor metabolic disorders (General)  Random subjects (General)  Random subjects (General)  Subjects with CAD and controls  (Hospital/trial)  Patients with stroke (Hospital/trial)  Patients with minor illnesses and local community-based residents free of neuro-imaging alterations and clinical history of stroke events (Hospital/trial)  Healthy control subjects (Hospital/trial)  Subjects with MetS (Hospital/trial)  Nondrinkers without atherosclerosis, CAD and diabetes (General)  Drinkers without atherosclerosis, CAD and diabetes (General)  Chinese healthy young subjects  (General)  Healthy controls (Hospital/trial)  Type 2 diabetes (Hospital/trial)  Type 2 diabetic with CHD (Hospital/trial)  Subjects in SY,SE,TE groups  (Hospital/trial)  Non-diabetic patients from Plymouth Early Bird Study (General)  Non-diabetic patients from Pune Children Study (General)  Hypertriglyceridemic patients  (Hospital/trial)  Yemenite from Jewish Israli cohort (General)  Sephardic from Jewish Israli cohort (General)  Ashkenazi from Jewish Israli cohort (General)  Normal control subjects (Hospital/trial)  Subjects with T2DM (Hospital/trial)  Patients with T2DM (Hospital/trial)  Control subjects (Hospital/trial)  Patients with CHD (Hospital/trial)  General subjects (Hospital/trial)  Patients with CHD and control subjects (Hospital/trial)  Control subjects (Hospital/trial)  Patients with T2DM (Hospital/trial)  Type 2 diabetic with CHD (Hospital/trial)  Patients with CHD (Hospital/trial)  Control subjects (Hospital/trial)  Patients with CHD (Hospital/trial)  Subjects with MetS  Control subjects | TC, TG, LDL-C, HDL-C  TC, TG, LDL-C, HDL-C  TC, TG, LDL-C, HDL-C  TC, TG, HDL-C  TC, TG, HDL-C  TG  TG  TG  TC, TG  TC, TG  TC, TG  TC, TG  TC, TG, LDL-C, HDL-C  TC, TG, HDL-C  TC, TG  TC, TG, LDL-C, HDL-C  TC, TG  TC, LDL-C, HDL-C  TC, LDL-C, HDL-C  TC, TG, LDL-C, HDL-C  TG, HDL-C  TC, TG, LDL-C, HDL-C  TC, TG, LDL-C, HDL-C  TC, TG, LDL-C, HDL-C  TC, TG, LDL-C, HDL-C  TG  TG  TG  TG  TG  TG  TG  TG  TG  TG  TC, TG  TC, TG, LDL-C, HDL-C  TC, TG, LDL-C, HDL-C  TC, TG, LDL-C, HDL-C  TG, HDL-C  TG, HDL-C  TC, TG, LDL-C, HDL-C  TC, TG, LDL-C, HDL-C  TC, TG, LDL-C, HDL-C  TC, TG, LDL-C, HDL-C  TC, TG, LDL-C, HDL-C  TC, TG, LDL-C, HDL-C  TC, LDL-C, HDL-C  TG, HDL-C  LDL-C, HDL-C  LDL-C, HDL-C  LDL-C, HDL-C  TG, HDL-C  TC, TG, LDL-C, HDL-C  TC, TG, LDL-C, HDL-C  TC, TG, LDL-C, HDL-C  TC, TG, LDL-C, HDL-C  TG, HDL-C  TG, HDL-C  TC,TG, LDL-C, HDL-C  TC, TG, LDL-C, HDL-C  TC, TG, LDL-C, HDL-C  TG, HDL-C  TG, HDL-C  TC, LDL-C, HDL-C  TC, LDL-C, HDL-C  TC,TG, HDL-C  TC, TG, LDL-C, HDL-C  TC, TG, LDL-C, HDL-C  TC, TG, LDL-C, HDL-C  TC,TG, LDL-C,HDL-C  TC, TG, LDL-C, HDL-C  TC, TG, LDL-C, HDL-C  TG,HDL-C  TG  TG  TG  TC,TG, LDL-C,HDL-C  TC,TG, LDL-C,HDL-C    TC,TG, LDL-C,HDL-C  TC,TG, LDL-C,HDL-C  TC,TG, LDL-C,HDL-C  TC,TG, HDL-C  TC,TG, LDL-C,HDL-C  TC,TG, LDL-C,HDL-C  TC,TG, LDL-C,HDL-C  TC,TG, LDL-C,HDL-C  TC,TG, LDL-C,HDL-C  TC, TG, LDL-C, HDL-C  TC, TG, LDL-C, HDL-C  TC,TG, LDL-C,HDL-C  TC,TG, LDL-C,HDL-C |

Abbreviations: NA, not available; CAD,coronary artery disease; CHD, coronary heart disease; MetS, metabolic syndrome; T2DM,

Type 2 Diabetes Mellitus; MONICA study, Multinational Monitoring of Trends and Determinants in Cardiovascular Diseases

study; M, male; F, female.
