# Supplementary material for: Effects of APOA5 −1131T>C (rs662799) on Fasting Plasma Lipids and Risk of Metabolic Syndrome: Evidence from a Case-Control Study in China and a Meta-Analysis
Source: PLoS One. 2013 Feb 28;8(2):e56216. doi: 10.1371/journal.pone.0056216 (PMC3585417; doi:10.1371/journal.pone.0056216)
Supplement: Table S2 — Plasma lipid levels by genotypes of individual studies included in the meta-analysis for the −1131T>C. (DOC) [file pone.0056216.s008.doc]

**Table S2. Plasma lipid levels by genotypes of individual studies included in the meta-analysis for the *-1131T>C.***

| Plasma lipid levels by genotypes of individual studies included in the meta-analysis for the *-1131T>C.* First author, year | Genotype | | | TC ^e^ | | TG ^e^ | | LDL-C ^e^ | | HDL-C ^e^ | |
| --- | --- | --- | --- | --- | --- | --- | --- | --- | --- | --- | --- |
|  | TC/CC | TT | Total | TC/CC | TT | TC/CC | TT | TC/CC | TT | TC/CC | TT |
| Lai 1, 2003^bc^ [12]  Lai 2, 2003 [12]  Lai 3, 2003 [12]  Chaaba 1, 2005 [27]  Chaaba 2, 2005 [27]  Járomi 1, 2010 [28]  Járomi 2, 2010 [28]  Chien, 2008^a^ [29]  Maasz 1, 2007 [30]  Maasz 2, 2007^a^ [30]  Maasz 1, 2008 [61]  Maasz 2, 2008 [61]  Charriere, 2008 [31]  Kisfali 1, 2010 [32]  Kisfali 2, 2010 [32]  Martinelli, 2007 [33]  Talmud, 2004 [34]  Hubacek 1, 2008^c^ [35]  Hubacek 2, 2008 [35]  Mattei, 2009 [17]  Hahne, 2008 [36]  Sundl, 2007 [37]  Elosua, 2006 [38]  Szalai 1, 2004^c^ [39]  Szalai 2, 2004^b^ [39]  Hodoglugil 1, 2006 [40]  Hodoglugil 2, 2006 [40]  Hodoglugil 3, 2006 [40]  Hodoglugil 4, 2006 [40]  Pennacchio 1, 2002 [6]  Pennacchio 2, 2002 [6]  Pennacchio 3, 2002^a^ [6]  Pennacchio 4, 2002 [6]  Pennacchio 5, 2002 [6]  Pennacchio 6, 2002 [6]  Huang, 2008 [7]  Girona, 2008 [41]  Jang 1, 2009 [42]  Jang 2, 2009 [42]  Yamada 1, 2007^a^ [43]  Yamada 1, 2007 [43]  Yamada 2, 2007^ab^ [43]  Li 1, 2008^a^ [44]  Li 2, 2008 [44]  Li 2, 2008 [44]  Hsu, 2006 [45]  Bi 1, 2004 [46]  Bi 2, 2004^a^ [46]  Baum, 2003 [8]  Hsu, 2008 [14]  Baum 1, 2007 [47]  Baum 2, 2007 [47]  Baum 3, 2007 [47]  Vasilopoulos, 2010 [16]  Olano-Martin, 2008 [48]  Jang, 2004 [49]  Aouizerat, 2003 [9]  Li, 2004^a^ [50]  Komurcu-Bayrak 1, 2008 [51]  Komurcu-Bayrak 1, 2008 [51]  Komurcu-Bayrak 2, 2008 [51]  Komurcu-Bayrak 2, 2008 [51]  Lee, 2004 [52]  Lee, 2004 [52]  Li 1, 2011 [53]  Li 2, 2011^b^ [53]  Yamada 1, 2007 [13]  Yamada 2, 2007 [13]  Yin 1, 2011 [54]  Yin 2, 2011 [54]  Lin, 2011 [55]  Yan 1, 2005 [55]  Yan 2, 2005^a^ [56]  Yan 3, 2005^a^ [56]  Song, 2012 [57]  Chandak 1, 2006 [58]  Chandak 2, 2006 [58]  Jang, 2010 [10]  Ken-Dror 1, 2010 [59]  Ken-Dror 2, 2010 [59]  Ken-Dror 3, 2010 [59]  Qiao 1, 2008 [64]  Qiao 2, 2008 [64]  Zhu, 2007 [65]  Tang, 2005 [71]  Qiu, 2007 [66]  Wang, 2010 [67]  Li, 2007 [68]  Cheng 1, 2007 ^b^ [69]  Cheng 2, 2007^a^ [69]  Cheng 3, 2007^a^ [69]  Yang, 2007 [70]  Liu 1, 2005 [62]  Liu 2, 2005^ab^ [62]  Xu 1, 2012  Xu 2, 2012^a^ | 1181  326  210  36  25  79  13  119  38  25  69  12  64  61  28  100  54  162  212  184  25  46  296  42  33  23  66  381  319  48  42  154  111  62  36  202  25  359  421  2146  2141  802  168  156  144  359  175  181  96  317  89  196  180  31  45  73  185  199  152  164  134  147  67  65  217  143  636  396  248  248  20  72  96  72  854  19  188  165  27  53  45  89  69  72  113  122  73  222  56  93  74  110  256  302  371  317 | 1235  334  298  100  76  434  159  163  163  185  309  119  336  282  256  516  242  957  1086  618  84  251  1971  174  277  208  505  1220  983  311  286  537  376  125  103  114  144  382  320  1640  1652  617  172  100  76  318  94  136  71  298  109  186  187  59  214  85  442  135  488  528  462  532  371  369  125  167  381  375  268  266  36  83  76  41  765  218  336  118  96  241  189  117  85  36  87  126  83  232  57  70  38  58  246  181  534  618 | 2416  660  508  136  101  513  172  282  201  210  378  131  400  343  284  616  296  1119  1298  802  109  297  2267  216  310  231  571  1601  1302  359  328  691  487  187  139  316  169  741  741  3786  3793  1419  340  256  220  677  269  317  167  615  198  382  367  90  259  158  627  334  640  692  596  679  438  434  342  310  1017  771  516  514  56  155  172  113  1619  237  524  283  123  294  234  206  154  108  200  248  156  454  113  163  112  168  502  483  905  935 | 5.49±1.20  5.69±1.55  5.66±1.20  5.17±1.07  4.53±0.99  -  -  -  5.84±1.48  4.71±1.95  5.77±1.25  4.79±0.69  5.33±0.89  5.51±1.11  5.18±1.06  226±44  5.26±0.63  5.77±1.00  5.94±1.18  4.70±1.13  -  5.06±0.878  212±41  6.18±0.75  5.59±0.50  -  -  -  -  -  -  -  -  -  -  187.66±41.10  4.79±0.63  198±34.36  172±40.77  -  -  -  4.79±0.93  4.96±0.94  5.25±1.12  206±43  5.35±0.86  5.03±1.06  5.19±0.78  -  -  -  -  -  5.64±0.84  191.7±37.04  264±84  178.30±43.74  -  -  -  -  5.15±1.23  -  5.32±1.43  4.56±1.60  -  -  4.56±1.06  4.90±0.84  149.3±25.6  4.93±0.95  5.11±0.90  4.93±1.15  187.9±35.4  202.6±23.5  164.0±35.2  -  -  -  -  4.76±0.80  4.89±0.95  4.93±0.50  4.50±1.14  4.85±1.01  4.71±0.85  4.79±0.84  4.97±0.95  5.14±0.88  4.92±1.13  4.05±0.92  5.22±0.82  5.51±0.80  5.09±1.03  4.41±0.81 | 5.39±1.41  5.57±1.64  5.54±1.38  5.17±1.15  4.61±1.32  -  -  -  5.30±1.53  5.02±1.50  5.79±1.23  5.22±0.98  5.42±1.11  5.30±1.06  5.42±1.00  222±41  5.18±0.73  5.72±1.04  5.78±1.14  4.77±1.09  -  4.82±0.932  205±37  6.10±0.79  5.52±0.62  -  -  -  -  -  -  -  -  -  -  187.25±36.65  4.68±0.79  196±36.55  172±40.96  -  -  -  4.69±1.08  4.84±0.98  5.02±1.04  202±36  5.31±1.10  4.98±0.98  5.22±0.93  -  -  -  -  -  5.77±1.02  188.5±34.76  264±77  165.90±37.96  -  -  -  -  5.18±0.96  -  5.15±1.22  4.47±1.42  -  -  4.48±0.92  4.68±0.92  150.4±36.2  4.73±0.88  5.00±0.87  4.56±0.88  188.5±32.7  189.8±36.9  156.6±36.7  -  -  -  -  4.82±0.83  4.73±1.05  4.83±0.72  4.70±1.21  4.78±1.19  4.68±0.73  4.71±1.04  4.72±0.93  4.98±0.88  4.54±0.88  4.00±0.92  5.03±0.78  5.52±0.76  5.03±0.93  4.36±0.76 | 1.58±1.48  1.95±1.87  1.93±1.08  2.62±1.59  1.75±1.07  1.92±0.80  1.84±0.43  102.3±39.8  3.22±2.65  2.10±0.95  2.14±1.08  2.00±1.04  2.24±1.06  2.90±1.55  1.66±0.54  186±104  1.85±1.00  -  -  1.95±1.82  1.87±1.51  1.21±0.582  172±135  2.22±0.62  1.82±0.79  166±80  142±79  174±120  122±88  135±97  213±175  100.32±76  150±158  167±101  252±186  132.58±92.00  2.14±1.29  155±91  164±106.91  1.78±1.66  -  1.75±1.04  1.61±0.25  1.78±0.38  1.94±0.35  161±155  2.41±2.07  1.85±1.31  -  1.79±1.45  -  -  -  150.1± 91.5  1.82±1.01  148.41±95.6  230±267  165.89±147.0  1.83±0.86  -  2.14±1.04  -  2.33±1.96  -  2.26±1.60  1.86±1.38  2.25±1.54  1.18±0.76  -  -  91.1±34.4  1.29±0.84  1.83±1.40  1.73±1.33  137.5±93.2  125.3±54.9  109.7±55.9  221.5±68.3  163±104.0  135±97.9  185±115.4  1.25±0.40  2.38±2.02  2.16±0.49  1.45±0..43  1.93±0.98  1.99±0.99  1.61±1.02  1.38±0.87  1.81±1.36  1.74±1.31  1.71±1.09  1.74±0.87  2.13±0.89  3.46±2.53  1.15±0.31 | 1.34±1.76  1.56±2.01  1.67±1.21  2.05±1.61  1.6±1.42  1.70±0.62  1.51±0.50  80.9±29.7  2.24±1.53  1.22±0.68  1.68±0.53  1.48±0.55  1.98±1.15  2.33±1.28  1.38±0.34  154±80  1.56±0.67  -  -  1.80±1.23  1.51±0.67  1.05±0.522  137±91  1.95±0.58  1.48±0.52  139±93  120±76  144±99  104±64  125±98  164±134  99±75  140±206  158±201  173±150  101.39±63.94  1.69±0.94  129±58.63  135±71.55  1.39±0.84  -  1.36±0.73  1.52±0.32  1.65±0.33  1.76±0.41  129±107  1.84±1.34  1.32±0.89  -  1.43±1.17  -  -  -  106.1± 81.3  1.59±0.73  113.7±60.9  198±198  112.72±79.1  1.56±0.88  -  1.86±1.07  -  1.81±1.35  -  2.06±1.25  1.76±1.42  1.96±1.03  1.05±0.54  -  -  67.7±45.5  0.92±0.57  1.18±0.87  1.16±0.43  120.9±69.7  95.3±56.1  91.5±56.8  207.9±60.3  126±124.2  138±84.1  137±99.5  1.10±0.30  1.74±1.02  1.91±0.42  1.26±0.40  1.74±0.93  1.54±0.73  1.29±0.74  0.91±0.60  1.17±0.90  1.09±0.34  1.53±0.71  1.39±0.78  1.73±0.89  2.93±1.99  1.15±0.32 | 3.48±1.18  3.77±1.39  3.83±1.19  -  -  -  -  -  -  -  -  -  3.39±0.85  -  -  151±40  -  3.68±0.98  3.78±1.12  2.73±0.95  -  2.89±0.652  129±35  3.89±0.95  3.47±0.65  -  -  -  -  -  -  -  -  -  -  -  2.68±0.76  117±32.38  96.3±39.51  -  -  -  2.59±0.42  2.81±0.44  3.05±0.52  124±37  3.37±0.96  3.00±0.83  3.19±0.78  -  3.24±0.85  3.26±0.93  3.59±1.18  -  3.58±0.83  112.0±28.6  173±68  94.29±28.2  -  -  -  -  3.42±0.82  -  3.43±0.98  3.17±0.87  -  -  2.43±0.70  2.47±0.66  -  3.11±0.85  3.22±0.79  3.15±0.99  106.1±30.6  123.9±24.0  101.5±32.5  -  -  -  -  2.77±0.97  2.69±0.98  2.42±0.36  2.66±0.87  2.88±0.82  -  2.75±0.69  3.00±0.76  3.25±0.77  3.16±0.98  2.31±0.74  3.07±0.96  3.20±0.88  2.28±0.76  2.00±0.58 | 3.37±1.05  3.72±1.46  3.75±1.21  -  -  -  -  -  -  -  -  -  3.53±0.90  -  -  148±37  -  3.65±0.95  3.61±1.04  2.80±0.84  -  2.68±0.728  128±32  3.91±0.95  3.59±0.55  -  -  -  -  -  -  -  -  -  -  -  2.83±0.69  120±33.62  99.3±36.31  -  -  -  2.51±0.49  2.67±0.48  2.94±0.46  123±31  3.30±0.90  3.07±0.60  3.35±0.84  -  2.92±0.81  3.23±0.82  3.73±1.22  -  3.72±1.02  115.3±29.2  175±62  88.73±24.4  -  -  -  -  3.61±0.96  -  3.37±0.78  3.11±0.82  -  -  2.37±0.71  2.35±0.72  -  3.11±0.82  3.17±0.69  3.03±0.82  107.3±28.5  115.5±35.4  96.8±33.0  -  -  -  -  2.87±0.88  2.75±1.08  2.45±0.50  2.78±0.99  2.84±1.01  -  2.70±0.65  2.69±0.69  3.16±0.71  3.03±0.83  2.24±0.72  3.01±0.89  3.22±0.83  2.17±0.75  1.93±0.58 | 1.34±0.31  1.21±0.46  1.13±0.39  0.83±0.34  0.83±0.26  -  -  -  -  -  -  -  0.97±0.24  1.22±0.31  -  48±15  -  1.21±0.37  1.47±0.37  1.15±0.33  1.39±0.38  1.33±0.296  50±16  1.29±0.14  1.28±0.16  -  -  -  -  -  -  -  -  -  -  -  1.15±0.31  49.2±12.50  43.4±11.25  -  1.32±0.47  1.45±0.37  1.54±0.31  1.25±0.33  1.06±0.27  51±15  1.12±0.33  1.34±0.36  1.15±0.39  1.40±0.39  1.51±0.44  1.37±0.39  1.14±0.31  46.9 ±8.9  1.24±0.38  50.0±17.0  55±19  53.20±14.69  -  1.17±0.26  -  1.02±0.24  -  0.87±0.24  1.32±0.36  1.38±0.30  1.12±0.32  1.61±0.32  1.97±0.49  2.13±0.52  57.8±15.4  1.33±0.36  1.35±0.36  1.07±0.31  45.4±11.9  54.2±10.0  40.8±11.8  42.3±11.6  -  -  -  1.40±0.59  1.14±0.36  1.36±0.32  1.19±0.22  1.06±0.24  1.37±0.06  1.26±0.38  1.27±0.35  1.36±0.36  1.06±0.31  1.04±0.24  1.65±0.85  1.03±0.45  1.42±0.42  1.63±0.36 | 1.42±0.35  1.28±0.55  1.14±0.35  0.90±0.32  0.92±0.25  -  -  -  -  -  -  -  1.08±0.29  1.24±0.50  -  51±15  -  1.25±0.34  1.51±0.37  1.16±0.33  1.52±0.38  1.33±0.308  51±16  1.30±0.16  1.26±0.18  -  -  -  -  -  -  -  -  -  -  -  1.09±0.28  50.5±12.31  45.2±10.91  -  1.40±0.42  1.56±0.39  1.62±0.38  1.34±0.32  1.13±0.32  54±19  1.20±0.29  1.34±0.36  1.28±0.34  1.45±0.35  1.64±0.36  1.40±0.36  1.20±0.35  50.1 ±12.8  1.34±0.44  50.1±10.1  59±24  55.31±15.72  -  1.26±0.23  -  1.03±0.23  -  0.95±0.19  1.36±0.30  1.42±0.31  1.19±0.40  1.67±0.35  1.98±0.41  2.15±0.47  63.0±13.6  1.48±0.34  1.44±0.31  1.06±0.22  46.9±11.7  55.8±14.0  41.4±12.8  43.4±9.0  -  -  -  1.43±0.48  1.14±0.32  1.44±0.37  1.21±0.25  1.05±0.32  1.40±0.09  1.30±0.38  1.46±0.35  1.45±0.32  1.07±0.21  1.04±0.27  1.69±0.81  1.28±0.48  1.46±0.44  1.67±0.41 |
| Total^d^ | 19939 31929 51868 | | | | | | | | | | |

Data were expressed as mean±SD.

^a^ Outlier studies for TG.

^b^ Outlier studies for HDL-C.

^c^ The study was found to deviate from HWE.

^d^ Among the 51457 subjects, 61.4% had the genotype TT, and 38.6% were carriers of -1131C allele.

^e^ Unified unit (mmol/L) were used in the studies included in this meta-analysis.

^f^ 28483, 41709, 26822, and 37078 subjects were included in comparing the difference in blood TC, TG, LDL-C, and HDL-C, respectively.
