# Supplementary material for: Effects of APOA5 −1131T>C (rs662799) on Fasting Plasma Lipids and Risk of Metabolic Syndrome: Evidence from a Case-Control Study in China and a Meta-Analysis
Source: PLoS One. 2013 Feb 28;8(2):e56216. doi: 10.1371/journal.pone.0056216 (PMC3585417; doi:10.1371/journal.pone.0056216)
Supplement: Table S3 — Studies included in the meta-analysis of the association of APOA5 −1131T>C with metabolic syndrome. (DOC) [file pone.0056216.s009.doc]

**Table S3. Studies included in the meta-analysis of the association of *APOA5* *-1131T>C* with metabolic syndrome.**

| First author, year, reference | Ethnicity | Study design | Genotyping method | MetS cases | | | | Controls | | | | Definition of MetS |
| --- | --- | --- | --- | --- | --- | --- | --- | --- | --- | --- | --- | --- |
|  |  |  |  | TT | TC | CC | Total | TT | TC | CC | Total |  |
| Yamada, 2007 [13] | East Asian | Hospital based | SAT-PCR | 381 | 483 | 153 | 1017 | 375 | 321 | 75 | 771 | NCEP ATP III |
| Mattei, 2009^g^ [17] | Other | Population based | TaqMan-PCR | 409 | 109 | 8 | 526 | 199 | 61 | 4 | 264 | NCEP ATP III |
| MAáSZ, 2007 [30] | Caucasian | Hospital based | PCR-RFLP | 163 | 32 | 6 | 201 | 185 | 24 | 1 | 210 | NCEP ATP III |
| Grallert 1, 2007 [18] | Caucasian | Population based | MALDI-TOF | 483 | 72 | 6 | 561 | 661 | 99 | 6 | 766 | NCEP ATP III |
| Grallert 2, 2007 [18] | Caucasian | Population based | MALDI-TOF | 401 | 66 | 3 | 470 | 1061 | 143 | 3 | 1207 | NCEP ATP III |
| Komurcu-Bayrak, 2008 [51] | Other | Population based | TaqMan-PCR | 550 | 174 | 16 | 740 | 642 | 171 | 11 | 824 | NCEP ATP III |
| Hsu, 2008 [14] | East Asian | Population based | PCR-RMGE | 44 | 56 | 15 | 115 | 254 | 215 | 31 | 500 | NCEP ATP III |
| Ong, 2011 [15] | East Asian | Population based | MassARRAY | 332 | 301 | 87 | 720 | 1340 | 1015 | 207 | 2562 | IDF |
| Niculescu, 2010 [19] | Caucasian | Population based | PCR-RFLP | 157 | 18 | 13 | 188 | 74 | 11 | 6 | 91 | NCEP ATP III |
| Vasilopoulos, 2011^g^ [16] | Caucasian | Hospital based | PCR-RFLP | 14 | 15 | 1 | 30 | 45 | 15 | 0 | 60 | NCEP ATP III |
| Dai, 2011^cg^ [63] | East Asian | Hospital based | PCR-RFLP | 53 | 34 | 13 | 100 | 74 | 19 | 7 | 100 | IDF |
| Xu, 2012 | East Asian | Population based | Illumina | 534 | 304 | 67 | 905 | 618 | 276 | 41 | 935 | IDF |

^c^ The study was found to deviate from HWE in control subjects.

^g^ Outlier studies for MetS.
