# Supplementary material for: Effects of APOA5 −1131T>C (rs662799) on Fasting Plasma Lipids and Risk of Metabolic Syndrome: Evidence from a Case-Control Study in China and a Meta-Analysis
Source: PLoS One. 2013 Feb 28;8(2):e56216. doi: 10.1371/journal.pone.0056216 (PMC3585417; doi:10.1371/journal.pone.0056216)
Supplement: Table S4 — Subgroup analysis in a meta-analysis of the effects of APOA5 −1131T>C on fasting plasma lipids and metabolic syndrome risk. (DOC) [file pone.0056216.s010.doc]

**Table S4. Subgroup analysis in a meta-analysis of the effects of *APOA5 -1131T>C* on fasting plasma lipids and metabolic syndrome risk.**

| Groups | Studies (n) | *I^2^* (%) | Q test *P* value | WMD (95% CI) | *P* |
| --- | --- | --- | --- | --- | --- |
| **TC** |  |  |  |  |  |
| Lipid-lowering medicine administer |  |  |  |  |  |
| Never use | 19 | 8.9 | 0.347 | 0.10 (0.04, 0.15) | 0.00032 |
| Not reported | 40 | 34.3 | 0.020 | 0.12 (0.10, 0.15) | 1.90×10^-7^ |
| Ethnicity |  |  |  |  |  |
| European | 18 | 20.8 | 0.206 | 0.10 (0.04, 0.15) | 0.001 |
| East Asian | 37 | 0.0 | 0.671 | 0.08 (0.05, 0.11) | 1.11×10^-6^ |
| Other | 8 | 29.5 | 0.192 | 0.06 (-0.04, 0.16) | 0.225 |
| Sex |  |  |  |  |  |
| Man | 5 | 0.0 | 0.728 | 0.08 (-0.02, 0.18) | 0.135 |
| Woman | 2 | 5.6 | 0.303 | 0.10 (-0.04, 0.25) | 0.151 |
| Not reported separately | 56 | 4.8 | 0.374 | 0.08 (0.05, 0.11) | 1.60×10^-8^ |
| Heath status |  |  |  |  |  |
| Healthy | 13 | 16.6 | 0.277 | 0.12 (0.05, 0.20) | 0.001 |
| MetS | 3 | 43.4 | 0.171 | 0.17 (-0.05, 0.38) | 0.076 |
| CHD | 9 | 0.0 | 0.549 | 0.05 (-0.02, 0.13) | 0.143 |
| Type 2 diabetes | 11 | 0.0 | 0.705 | 0.13 (0.04, 0.22) | 0.004 |
| Sample size |  |  |  |  |  |
| Large (≥500) | 19 | 8.5 | 0.351 | 0.09 (0.05, 0.12) | 1.23×10^-6^ |
| Small (<500) | 44 | 0.0 | 0.549 | 0.07 (0.03, 0.11) | 0.00034 |
| **TG** |  |  |  |  |  |
| Lipid-lowering medicine administer |  |  |  |  |  |
| Never use | 28 | 33.6 | 0.044 | 0.31 (0.26, 0.37) | 1.27×10^-33^ |
| Not reported | 48 | 78.0 | 0.00001 | 0.32 (0.18, 0.47) | 8.73×10^-6^ |
| Ethnicity |  |  |  |  |  |
| European | 24 | 0.0 | 0.492 | 0.32 (0.27, 0.38) | 1.92×10^-28^ |
| East Asian | 41 | 83.5 | <0.00001 | 0.31 (0.25, 0.36) | 2.22×10^-25^ |
| Other | 19 | 14.9 | 0.272 | 0.23 (0.18, 0.28) | 6.52×10^-19^ |
| Sex |  |  |  |  |  |
| Man | 9 | 0.0 | 0.588 | 0.29 (0.20, 0.37) | 1.81×10^-11^ |
| Woman | 7 | 34.5 | 0.165 | 0.20 (0.14, 0.27) | 8.56×10^-9^ |
| Not reported separately | 68 | 75.7 | 0.00001 | 0.31 (0.27, 0.36) | 7.16×10^-42^ |
| Heath status |  |  |  |  |  |
| Healthy | 13 | 75.6 | 0.00001 | 0.29 (0.20, 0.38) | 6.36×10^-10^ |
| MetS | 4 | 37.0 | 0.190 | 0.38 (0.25, 0.51) | 1.95×10^-8^ |
| CHD | 9 | 24.6 | 0.225 | 0.35 (0.27, 0.42) | 1.42×10^-19^ |
| Type 2 diabetes | 9 | 58.6 | 0.013 | 0.31 (0.19, 0.43) | 6.51×10^-7^ |
| Sample size |  |  |  |  |  |
| Large (≥500) | 27 | 79.8 | <0.0001 | 0.27 (0.21, 0.33) | 2.14×10^-19^ |
| Small (<500) | 57 | 55.7 | <0.0001 | 0.32 (0.27, 0.37) | 1.56×10^-37^ |
| **LDL-C** |  |  |  |  |  |
| Lipid-lowering medicine administer |  |  |  |  |  |
| Never use | 19 | 20.1 | 0.209 | 0.04 (-0.01, 0.09) | 0.082 |
| Not reported | 29 | 13.7 | 0.256 | 0.06 (0.03, 0.09) | 0.00006 |
| Ethnicity |  |  |  |  |  |
| European | 11 | 37.8 | 0.098 | 0.02 (-0.06, 0.10) | 0.611 |
| East Asian | 37 | 18.4 | 0.166 | 0.05 (0.02, 0.08) | 0.001 |
| Other | 6 | 0.0 | 0.433 | 0.02 (-0.06, 0.09) | 0.697 |
| Sex |  |  |  |  |  |
| Man | 4 | 49.0 | 0.117 | 0.01 (-0.14, 0.16) | 0.878 |
| Woman | 1 | - | - | 0.17 (0.01, 0.33) | 0.041 |
| Not reported separately | 49 | 16.6 | 0.163 | 0.04 (0.01, 0.07) | 0.002 |
| Heath status |  |  |  |  |  |
| Healthy | 11 | 37.3 | 0.101 | 0.04 (-0.03, 0.10) | 0.260 |
| MetS | 1 | - | - | 0.11 (0.01, 0.21) | 0.031 |
| CHD | 8 | 0.0 | 0.872 | 0.00 (-0.07, 0.08) | 0.938 |
| Type 2 diabetes | 12 | 1.8 | 0.426 | 0.05 (-0.01, 0.10) | 0.121 |
| Sample size |  |  |  |  |  |
| Large (≥500) | 18 | 19.3 | 0.233 | 0.04 (0.01, 0.08) | 0.016 |
| Small (<500) | 36 | 22.0 | 0.122 | 0.04 (0.00, 0.08) | 0.049 |
| **HDL-C** |  |  |  |  |  |
| Lipid-lowering medicine administer |  |  |  |  |  |
| Never use | 27 | 52.4 | 0.001 | -0.05 (-0.07, -0.03) | 9.07×10^-7^ |
| Not reported | 35 | 18.7 | 0.167 | -0.05 (-0.06, -0.04) | 3.03×10^-27^ |
| Ethnicity |  |  |  |  |  |
| European | 14 | 24.7 | 0.188 | -0.04 (-0.06, -0.02) | 0.001 |
| East Asian | 45 | 43.0 | 0.001 | -0.06 (-0.07, -0.04) | 6.83×10^-17^ |
| Other | 10 | 37.2 | 0.111 | -0.05 (-0.07, -0.02) | 0.001 |
| Sex |  |  |  |  |  |
| Man | 5 | 10.3 | 0.347 | -0.03 (-0.06, 0.01) | 0.115 |
| Woman | 2 | 48.6 | 0.163 | -0.07 (-0.12, -0.02) | 0.007 |
| Not reported separately | 62 | 40.3 | 0.001 | -0.05 (-0.06, -0.04) | 1.80×10^-19^ |
| Heath status |  |  |  |  |  |
| Healthy | 14 | 0.0 | 0.486 | -0.05 (-0.07, -0.03) | 0.00001 |
| MetS | 3 | 0.0 | 0.565 | -0.05 (-0.09, -0.02) | 0.002 |
| CHD | 8 | 76.4 | 0.00011 | -0.05 (-0.10, 0.00) | 0.071 |
| Type 2 diabetes | 13 | 0.0 | 0.490 | -0.06 (-0.08, -0.03) | 0.00001 |
| Sample size |  |  |  |  |  |
| Large (≥500) | 26 | 29.3 | 0.082 | -0.05 (-0.07, -0.04) | 9.07×10^-19^ |
| Small (<500) | 43 | 43.5 | 0.002 | -0.05 (-0.07, -0.03) | 6.22×10^-9^ |
| **MetS** |  |  |  |  |  |
| Ethnicity |  |  |  |  |  |
| East Asian | 5 | 43.6 | 0.131 | 1.43 (1.29, 1.58) | 2.5×10^-12^ |
| European | 5 | 54.3 | 0.068 | 1.30 (0.94, 1.78) | 0.109 |
| Other | 2 | 58.3 | 0.122 | 1.10 (0.91, 1.34) | 0.333 |
| Study design |  |  |  |  |  |
| Population based | 8 | 26.5 | 0.217 | 1.23 (1.13, 1.35) | 6.2×10^-6^ |
| Hospital based | 4 | 32.9 | 0.215 | 1.70 (1.44, 2.01) | 7.1×10^-10^ |

Abbreviations: WMD, weighted mean difference; HWE, Hardy-Weinberg Equilibrium; TC: total cholesterol ; TG: triglycerides; LDL-C: low-density lipoprotein-cholesterol; HDL-C: high-density lipoprotein-cholesterol; MetS, metabolism syndrome; CHD, coronary heart disease.

| Groups | Studies (n) | *I^2^* (%) | Q test *P* value | WMD (95% CI) | *P* |
| --- | --- | --- | --- | --- | --- |
| **TC** |  |  |  |  |  |
| All | 63 | 0.0 | 0.512 | 0.08 (0.05, 0.10) | 1.74×10^-9^ |
| All in HWE | 60 | 2.8 | 0.413 | 0.08 (0.05, 0.11) | 1.35×10^-8^ |
| **TG** |  |  |  |  |  |
| All | 84 | 68.0 | 0.00001 | 0.30 (0.26, 0.33) | 1.87×10^-55^ |
| All in HWE | 82 | 68.7 | 0.00001 | 0.30 (0.26, 0.34) | 2.85×10^-53^ |
| **LDL-C** |  |  |  |  |  |
| All | 54 | 19.7 | 0.109 | 0.04 (0.02, 0.07) | 0.002 |
| All in HWE | 51 | 21.3 | 0.095 | 0.04 (0.01, 0.07) | 0.006 |
| **HDL-C** |  |  |  |  |  |
| All | 69 | 39.6 | 0.001 | -0.05 (-0.06, -0.04) | 1.88×10^-21^ |
| All in HWE | 66 | 38.0 | 0.001 | -0.05 (-0.06, -0.04) | 5.94×10^-20^ |
| **MetS** |  |  |  |  |  |
| All | 12 | 55.4 | 0.010 | 1.33 (1.16, 1.53) | 0.00004 |
| All in HWE | 11 | 50.2 | 0.029 | 1.30 (1.14, 1.47) | 0.00007 |
