# Supplementary material for: Effects of APOA5 −1131T>C (rs662799) on Fasting Plasma Lipids and Risk of Metabolic Syndrome: Evidence from a Case-Control Study in China and a Meta-Analysis
Source: PLoS One. 2013 Feb 28;8(2):e56216. doi: 10.1371/journal.pone.0056216 (PMC3585417; doi:10.1371/journal.pone.0056216)
Supplement: Table S5 — Meta-analysis of the effects of APOA5 −1131T>C on fasting plasma lipids and metabolic syndrome risk (excluding the outlier studies). (DOC) [file pone.0056216.s011.doc]

**Table S5. Meta-analysis of the effects of *APOA5 -1131T>C* on fasting plasma lipids and metabolic syndrome**

**risk (excluding the outlier studies).**

| Groups | Studies (n) | *I^2^* (%) | Q test *P* value | WMD (95%CI) | *P* |
| --- | --- | --- | --- | --- | --- |
| **TG** |  |  |  |  |  |
| All | 71 | 12.0 | 0.204 | 0.25 (0.23, 0.27) | <0.00001 |
| All in HWE | 69 | 14.5 | 0.161 | 0.25 (0.23, 0.27) | <0.00001 |
| Lipid-lowering medicine administer |  |  |  |  |  |
| Never use | 23 | 0 | 0.462 | 0.25 (0.21, 0.29) | <0.00001 |
| Not reported | 40 | 23.3 | 0.097 | 0.25 (0.22, 0.28) | <0.00001 |
| Ethnicity |  |  |  |  |  |
| European | 23 | 0.0 | 0.893 | 0.31 (0.25, 0.37) | <0.00001 |
| East Asian | 30 | 37.4 | 0.022 | 0.25 (0.22, 0.29) | <0.00001 |
| Other | 18 | 0.0 | 0.683 | 0.25 (0.20, 0.30) | <0.00001 |
| Sex |  |  |  |  |  |
| Man | 9 | 0.0 | 0.588 | 0.29 (0.20, 0.37) | <0.00001 |
| Woman | 6 | 0.0 | 0.745 | 0.25 (0.17, 0.32) | 0.00002 |
| Not reported separately | 56 | 20.8 | 0.105 | 0.25 (0.22, 0.27) | <0.00001 |
| Heath status |  |  |  |  |  |
| Healthy | 8 | 1.6 | 0.417 | 0.22 (0.15, 0.29) | <0.00001 |
| MetS | 4 | 0.0 | 0.420 | 0.38 (0.26, 0.50) | <0.00001 |
| CHD | 6 | 0.0 | 0.634 | 0.29 (0.20, 0.38) | <0.00001 |
| Type 2 diabetes | 7 | 21.9 | 0.262 | 0.20 (0.12, 0.28) | <0.00001 |
| Sample size |  |  |  |  |  |
| Large (≥500) | 23 | 5.4 | 0.387 | 0.26 (0.22, 0.29) | <0.00001 |
| Small (<500) | 48 | 16.2 | 0.171 | 0.24 (0.21, 0.28) | <0.00001 |
| **HDL-C** |  |  |  |  |  |
| All | 63 | 0.0 | 0.516 | -0.05 (-0.06, -0.04) | <0.00001 |
| All in HWE | 61 | 0.0 | 0.526 | -0.05 (-0.06, -0.04) | <0.00001 |
| Lipid-lowering medicine administer |  |  |  |  |  |
| Never use | 24 | 10.6 | 0.314 | -0.05 (-0.06, -0.03) | <0.00001 |
| Not reported | 32 | 0.4 | 0.460 | -0.05 (-0.06, -0.04) | <0.00001 |
| Ethnicity |  |  |  |  |  |
| European | 13 | 8.9 | 0.357 | -0.04 (-0.07, -0.02) | 0.00005 |
| East Asian | 40 | 0.0 | 0.736 | -0.05 (-0.06, -0.04) | <0.00001 |
| Other | 10 | 37.2 | 0.111 | -0.05 (-0.07, -0.02) | 0.001 |
| Sex |  |  |  |  |  |
| Man | 5 | 10.3 | 0.347 | -0.03 (-0.06, 0.01) | 0.101 |
| Woman | 2 | 48.6 | 0.163 | -0.07 (-0.10, -0.04) | 0.007 |
| Not reported separately | 56 | 0.0 | 0.633 | -0.05 (-0.06, -0.04) | <0.00001 |
| Heath status |  |  |  |  |  |
| Healthy | 15 | 13.0 | 0.308 | -0.06 (-0.07, -0.04) | <0.00001 |
| MetS | 3 | 0.0 | 0.565 | -0.05 (-0.09, -0.02) | 0.002 |
| CHD | 7 | 0.0 | 0.464 | -0.03 (-0.05, -0.00) | 0.034 |
| Type 2 diabetes | 11 | 0.0 | 0.587 | -0.07 (-0.09, -0.04) | <0.00001 |
| Sample size |  |  |  |  |  |
| Large (≥500) | 24 | 1.1 | 0.445 | -0.05 (-0.06, -0.04) | <0.00001 |
| Small (<500) | 39 | 0.0 | 0.507 | -0.04 (-0.06, -0.03) | <0.00001 |
| **MetS** |  |  |  |  |  |
| All | 9 | 24.6 | 0.225 | 1.33 (1.23, 1.45) | <0.00001 |
| All in HWE | 9 | 24.6 | 0.225 | 1.33 (1.23, 1.45) | <0.00001 |
| Ethnicity |  |  |  |  |  |
| East Asian | 4 | 13.2 | 0.327 | 1.40 (1.27, 1.55) | <0.00001 |
| European | 4 | 19.6 | 0.292 | 1.16 (0.96, 1.42) | 0.126 |
| Other | 1 | - | - | 1.22 (0.97, 1.54) | 0.096 |
| Study design |  |  |  |  |  |
| Population based | 7 | 0.0 | 0.480 | 1.27(1.15, 1.39) | <0.00001 |
| Hospital based | 2 | 0.0 | 0.767 | 1.60(1.33, 1.91) | <0.00001 |
